# PD-L1 autoregulation promotes the proliferation, migration and invasion of glioblastoma cells via GP130/JAK2/STAT3/IRAK2/IL6 signaling pathway

**DOI:** 10.1038/s41598-025-19169-2

**Published:** 2025-10-08

**Authors:** Xin Xie, Jingdi Bai, Yuhui Peng, Pingping Chen, Zhuxue Zhang, Daogui Fan, Feng Zhou, Yuan Yuan, Jiyun Liu, Yu Zhou, Qin Hong, Jianjun Hu, Juanjuan Duan, Wenli Fu, Ting Zhang, Hongmei Zeng, Jie Deng, Yangting Dong, Ying Liu, Xiaolan Qi, Wei Hong, Yan He, Chunlin Zhang, Wenfeng Yu, Qintian Yang, Wei Yi, Hua Bai, Qifang Zhang

**Affiliations:** 1 Key Laboratory of Endemic and Ethnic Diseases, Ministry of Education, School of Basic Medical Science, Guizhou Medical University, Guiyang, 550004 Guizhou China; 2 Department of Pathology, Guizhou Provincial People’s Hospital, Guizhou University, Guiyang, 550002 Guizhou China; 3 The Second Hospital of Tianjin Medical University, Tianjin, 300211 China; 4 Guizhou Provincial Staff Hospital, Guiyang, 550025 Guizhou China; 5 The Second People’s Hospital of Guiyang, Guiyang, 550081 Guizhou China; 6 Key Laboratory of Human Brain Bank for Functions and Diseases of Department of Education of Guizhou Province, College of Basic Medical, Guizhou Medical University, Guiyang, 550025 China; 7 The Second Affiliated Hospital of Guizhou, University of Traditional Chinese Medicine, Guiyang, 550002 Guizhou China; 8 Department of Neurology, Wulong District People’s Hospital, Wulong, Chongqing, 408500 China; 9 Medical Laboratory Center, The Third Affiliated Hospital of Guizhou Medical University, Duyun, 558000 Guizhou China; 10 Guizhou Provincial Key Laboratory of Pathogenesis and Drug Research on Common Chronic Diseases, Guiyang, 550004 Guizhou China

**Keywords:** Glioblastoma, PD-L1, GP130, STAT3, IRAK2, IL6, CNS cancer, CNS cancer, CNS cancer, Cell invasion, Mechanisms of disease

## Abstract

Glioblastoma (GBM) cells mediate immunosuppressive microenvironment, leading to poor response to programmed death-ligand 1(PD-L1) inhibitor therapy. How PD-L1 in GBM cells confers to GBM immunosuppressive microenvironment remains unclear. Here, we demonstrate that PD-L1 activated GP130/JAK2/STAT3/IRAK2/NFκB/IL6 pathway. We showed that PD-L1 was elevated in human primary GBM and promoted GBM cell viability, migration and invasion via activating janus kinase 2(JAK2) by interacting with glycoprotein 130(GP130) and PD-1 in GBM cells, leading to the activation of signal transducer and activator of transcription 3 (STAT3), a master that renders GBM immunosuppression. Overexpressing STAT3 reversed the effect of silencing PD-L1-mediated cell viability, proliferation, migration and invasion. STAT3 promoted interleukin-1 receptor-associated kinase 2(IRAK2) transcription. IRAK2 overexpression rescued the effect of silencing STAT3-mediated cell viability, proliferation, migration and invasion. Additionally, IRAK2 facilitated interleukin-6 (IL6) expression via activating nuclear factor kappa-B (NFκB). Our results indicate that PD-L1/GP130/JAK2/STAT3/IRAK2/NFκB/IL6 axis promotes GBM cell viability, proliferation, migration and invasion via interacting with GP130 and PD-1 in GBM cells. Additionally, STAT3 transcriptionally promoted PD-L1 expression, thus forming loops to lead to PD-L1 autoregulation. IL6 positively correlated with GBM-associated macrophages, myeloid-derived suppressor cells and the inhibition of dendritic cells. Our study suggests that PD-L1 autoregulation may create immunosuppressive GBM via activating GP130/JAK2/STAT3/IRAK2/IL-6 pathway.

**Supplementary Information:**

The online version contains supplementary material available at 10.1038/s41598-025-19169-2.

## Introduction

Glioblastoma multiforme (GBM) is the most aggressive brain cancer. Although advanced progress in cancer therapy has been made, patients with GBM have median survival times of approximately 14 months after the histological diagnosis^1,2^. Immunotherapy provides an opportunity for GMB as a potential therapeutic option^3^. However, there are almost no effective programmed death-ligand 1(PD-L1) / programmed death-1(PD-1) blockade therapies for the patients with GBM in most clinical trials, which is mainly due to the fact that GBM exists in highly immunosuppressive microenvironment^4–6^. Therefore, it is urgent to investigate the factors contributing to intrinsic immunosuppressive GBM microenvironment.

GBM cells have been shown to promote immunosuppressive milieu via multiple immune checkpoints and immune-inhibitory cytokines^7^. GBM cells can induce local microenvironmental changes through signal transduction and genetic alterations^8^. PD-L1 /PD-1 signaling pathway is one of the most promising immune checkpoints that effectively mediate antitumor effect of T cells. PD-L1 is highly expressed in GBM tissues^9^. Highly expressed PD-L1 correlates with worse survival of patients with GBM^10^ and contributes to immunosuppression in primary GBM^11^. Targeting PD-L1 by ACT001 has been reported to inhibit PD-L1 expression via inhibiting STAT3 activity and enhance anti-tumor immune response in GBM-bearing mice^12^, indicating that inhibiting PD-L1 in GBM cells overcome immunosuppression. However, anti-PD-L1/PD1 monotherapy has shown limited efficacy for GBM in clinical trials such as NCT03750071,NCT03047473 and NCT03233152, etc^5^. This partly stems from the fact that cancer cells express some factors that inhibit PD-L1/PD-1 blockade therapy^13^. For instance, vascular endothelial growth factor (VEGF) signaling contributes to tumor suppression^14^, therefore anti-PD-1/VEGF-A antibody was developed for patients with advanced solid tumors^15^. Therefore, understanding intracellular PD-L1 signaling and the factors that mediates PD-L1 expression in GBM cells holds a promising opportunity for developing effective PD-L1/PD-1 blockade therapy.

In this study, we demonstrated a feedback pathway where PD-L1 can activate GP130/JAK2/STAT3/IRAK2/IL6 via interacting GP130 and PD-1 in GBM cells, and STAT3 promotes PD-L1 expression. IL6 is positively correlated GBM-associated macrophages, myeloid-derived suppressor cells and inhibition of dendritic cells in GBM. Our study suggests that developing a bispecific antibody anti-IL6 × PD-L1 might be an option for effective targeting PD-L1 signaling in GBM.

## Materials and methods

### Dataset for bioinformatics analyses, GBM cell lines, primary GBM samples and antibodies

GEPIA II (http://gepia2.cancer-pku.cn/)^16^ was used to analyze PD-L1 expression and survival curve from TCGA database between normal and GBM. The correlation data were downloaded from TCGA-GBM dataset in cbioportal (https://www.cbioportal.org/). The correlation was plotted using Graphpadprism 10.0.

Human GBM cell lines T98 and U87 were purchased from the American Type Culture Collection (Rockville, MD, USA), and human normal glioblastoma cell line HA1800 cell line was purchased from ScienCell. The cells were cultured in dulbecco’s modified eagle medium (DMEM) containing 10% heat-inactivated fetal bovine serum (FBS), 100U/mL penicillin and 100 µg/mL streptomycin in a humidified atmosphere of 5% CO2 at 37 °C.

Eighty paraffin human GBM tissue samples and 20 Paraffin samples of adjacent tissues of GBM were obtained from Department of Pathology, Guizhou Provincial People’s Hospital. All patients were the first-time cases, and none of them had underlying diseases and received preoperative radiotherapy or chemotherapy. GBM was diagnosed by two senior pathologists in a double-blind manner. This study was approved by the medical Ethics Committee of the hospital. The specimens were conducted in accordance with the Declaration of Helsinki (IRB2024-131). The information for clinical samples was listed in supplementary Table 1. Antibodies were listed in supplementary Table 2.

### Serum PD-L1 measurement

Human serum PD-L1 was determined using Human PD-L1 CatchPoint^®^ SimpleStep ELISA^®^ Kit (ab278124) following the manufacture’ s instructions. After clot formation, blood samples were centrifuged at 2,000 x g for 10 min. Sera was collected. Each well /48 plate was added with 50 µL standard or sample, 50 µL Antibody Cocktail, sealed and incubated at room temperature for 1 h on a plate shaker set to 400 rpm., aspirated, washed three times with 350 µL 1X Wash Buffer, added with 100 µL of prepared CatchPoint HRP Development Solution and incubated for 10 min in the dark on a plate shaker set to 400 rpm. The plate was read at Ex/Cutoff/Em 530/570/590 nm under a microplate reader.

### Western blot

Cell lysates were subjected to sodium dodecyl sulfate-polyacrylamide gel electrophoresis (SDS-PAGE), transferred to polyvinylidene difluoride (PVDF) membrane. The membrane was blocked with 5% BSA for 1 h, and incubated with specific antibodies at 4 °C overnight. After washing, the membranes were incubated with horseradish peroxidase-labeled anti-rabbit, or anti-mouse antibody for 1 h at room temperature, then the protein bands were detected by an enhanced chemiluminescence detection system (ECL kit; Minipore), and the signals were visualized using Licor Odyssey Scanner. Signal intensity was quantified using Image J.

### Hematoxylin and eosin(H&E) staining and immunohistochemistry (IHC) staining

Paraffin-embedded GBM specimens were sectioned, deparaffinized and dewaxed. 

For H&E staining, the slides were dehydrated with gradient alcohol, rinsed with ddH_2_O, stained with hematoxylin solution (Solarbio, Cat. No. G1120), differentiated, stained with eosin solution, dehydrated, cleared and mounted with neutral resin. For IHC staining, the sections were dehydrated, immersed in Tris- Ethylene diamine tetraacetie acid (EDTA)-buffer (pH 9.0) and citrate buffer (pH 6.0) in a microwave at high power for 8 min for antigen retrieval. After cooling to room temperature, the slides were incubated at 3% H_2_O_2_ at room temperature for 10 min for blocking endogenous peroxidase activity, incubated with primary antibody at 4℃ overnight, the secondary antibody for 1 h. The color was developed by diaminobenzidine (DAB) for 3 min. Images were captured and analyzed under a LEICA DM500 microscope (LEICA, Germany).

### SiRNA transfection and cell counting kit 8 (CCK8) assay

siRNAs sequences were listed in supplementary Table 3. siRNAs were synthesized by Shanghai Genepharma Co., Ltd. (Shanghai, China). Cells were seeded in 60 mm dishes with medium (without antibiotics and FBS) and transfected with 50 nM siRNA against gene of interest or 50 nM negative control (siNC) using EntransterTM-R4000 Transfection Reagent (Engreen Biosystem) for 8 h, then supplemented with FBS. Cells were collected after 48 h of transfection. Silencing efficacy was verified by Western blot.

Cell viability was assayed using the CCK8 assay kit (Shanghai Yeasen Biotechnology Co., Ltd., Shanghai, China) following the manufacturer’s instructions. Briefly, 2000 cells/well were seeded in a well in 96-per well plate, incubated for 24, 48 and 72 h, respectively, then 10 µL CCK-8 solution was added, mixed, incubated for 2 h. Then optical density (OD) values were measured at 450 nm in a multifunctional plate-reader (Tecan, Switzerland).

### Colony formation

Cells were inoculated into a 6-well plate according to the density of 8 × 10^2^ cells/well, cultured for 12–14 days, washed with PBS, fixed with 4% paraformaldehyde for 30 min, stained with 0.25% crystal violet for 30 min and washed with PBS.

### EdU (5-Ethynyl-2′-deoxyuridine) incorporation

Edu incorporation into cells was assayed using BeyoClick™ EdU Cell Proliferation Kit with Alexa Fluor 555 (Beijing, China) following the instructions. Briefly, cells were seeded in a 96-well plate with a density of 1 × 10^4^ cells/well, incubated overnight. After EdU labeling, the cells were fixed, washed and permeabilized with 0.3% TritonX-100. Click reaction solution was added after cleaning, and 4’,6-diamidino-2-phenylindole (DAPI)dye was re-dyed after cleaning. The inverted fluorescence microscope Olympus IX51 (Olympus, Japan) was used to capture images. EDU positive rate (%) = number of positive cells/total cell number ×100%.

### Cell migration, invasion and wound healing

Cell invasion was assayed in a 24-well transwell chamber system (Corning, Cambridge, USA; pore size 8 μm) precoated with Matrigel (BD Biotechnology, USA). The lower chamber was filled with 20% FBS. 2.5 × 10^4^ cells were seeded in an upper chamber with a medium containing 5% FBS and cultured for 24 h. Cells on the lower surface were washed with PBS, fixed with 4% paraformaldehyde for 30 min, stained with crystal violet for 30 min. Cell migration was assayed as described above but without Matrigel. For wound healing capacity, cells were seeded at a density of 1 × 10^6^ cells/well in 6-well plates, incubated for overnight, wounded by scraping of the lines with a 200 µl pipette tip, washed with PBS and incubated in complete medium. The wounds were photographed under a microscope (Olympus, Japan).

### PDL-1 ~ GP130 Docking

The crystal structure of the PD-L1 and GP130 proteins were downloaded from the Protein Data Bank (PDB) database. PD-L1 and GP130 structures were processed by AutoDock (version 1.5.6). Docking PD-L1 with GP130 was performed using precise precision (XP) of the Ligand Docking module of the Schrödinger kit. Binding free energy between PD-L1 and GP130 was calculated using Molecular Mechanics/Generalized Born Surface Area (MM/GBSA) tool in Prime module of the Schrödinger kit. Protein-Ligand Interaction Profiler (PLIP) was used to analyze the binding interface of protein ligand complexes^8^. pyMOL 2.5 software was applied to supplement the interaction related details.

### Cellular thermal shift assay (CESTA)

CESTA is a common technique to validate the protein-ligand binding^17^. GBM cells were incubated with recombinant human PD-L1 (rPD-L1, Novoprotein, Cat. No.C315) or vehicle as control at 25 °C for 30 min on a microplate incubator shaker, then suspended in 1× PBS, heated for 3 min at five different temperatures, ranging from 45 °C to 65 °C, and then frozen in dry ice for 5 min. After repeated freeze-thaw cycles for cell lysis, GBM cells were centrifuged at 20,000 g for 20 min at 4 °C. The GP130 protein was determined by Western blot.

### Co-immunoprecipitation (Co-IP)

Protein A + G magnetic beads (MCE, Cat. No. HY-K0202) were used to hatch the indicated cell lysates for a whole night at 4 °C with specific primary antibodies. The proteins in the immunopreciptates were separated by Western blot.

### Immunofluorescence (IF) staining

IF protocol was performed as previously described^18^.The cells were fixed with 4% formaldehyde, incubated in 0.3% Triton X-100 (Sigma, USA) for 1 h at room temperature, blocked with 5% bovine serum albumin (BSA), incubated with anti-PD-L1, anti-GP130 diluted in PBS/1% BSA at 4 °C for overnight, stained with AF488-conjugated Goat Anti-Rabbit IgG(H + L) and AF594-conjugated Goat Anti-Rabbit IgG(H + L) secondary antibodies diluted in PBS/1% BSA for 60 min at room temperature. Images were captured under an inverted Olympus SpinSR10 Super-resolution Laser Scanning Confocal Microscope (Olympus, Japan).

### Dual-luciferase reporter assay activity assay

IRAK2 promoter and PD-L1 promoter were cloned into pGL4-luc to obtain pGL4-IRAK2-luc and pGL4-PD-L1-luc, respectively. U87 cells were co-transfected with pGL4-Basic-luc or pGL4-IRAK2-luc, or pGL4-PD-L1-luc, pcDNA3.1, pcDNA3.1-STAT3 or pRL-TK (control Renilla luciferase) using Lipofectamine 2000 (Life Technologies), respectively. Luciferase activity was analyzed using the dual-luciferase reporter assay activity assay (Promega) following the manufacturer’s protocol.

### Analysis of ChIP-seq peaks

ChIP-seq data of H3K4me3, K3K27ac and STAT3 were downloaded from Encyclopedia of DNA Elements (ENCODE, https://www.encodeproject.org) and Cistrome (http://www.cistrome.com/). Picard package was used to sort bam files and establish index for sorted bam files. MACS2 package was applied for peak calling. Integrative Genomics Viewer (IGV, version 2.19.2) was used to visualize ChIP peaks.

### Statistical analyses

Data were analyzed using GraphPad Prism 10.0 (GraphPad Software.Inc., San Diego, CA, USA) and presented as mean ± SD as indicated. Student’s t test was used to compare the differences between two groups. One-way ANOVA was used to compare the differences between more than two groups. **χ**^2^ test was used for the difference analysis of counting data, *P* < 0.05 was considered statistically significant. *P* values were indicated in the figures with asterisks: *P < 0.05, **P < 0.01, ***P < 0.001, and ****P < 0.0001; ns: not significant.

## Results

### PD-L1 expression was elevated in GBM and correlated with overall survival and disease-free survival

**Fig. 1:**
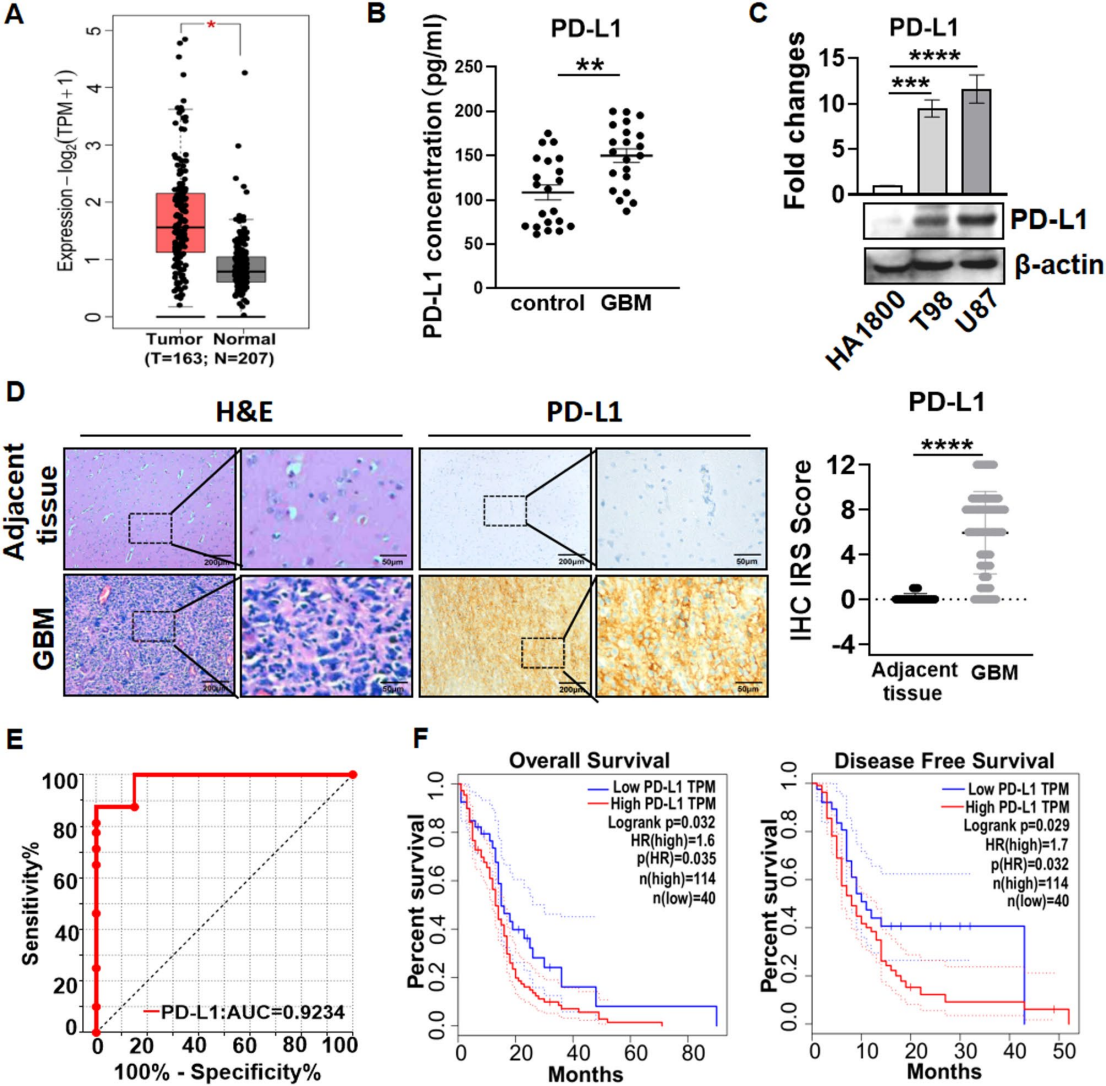
PD-L1 mRNA expression was elevated and positively associated with poor survival of patients with GBM. (**A**) Expression of PD-L1 RNA in human GBM tissues and normal brain tissue in TCGA-GBM dataset analyzed by using GEPIA online tool. (**B**) Serum PD-L1 expressions in healthy controls and GBM patients. (**C**) PD-L1 protein expressions in HA1800 and GBM cell lines. (**D**) Representative IHC staining images for PD-L1 in human primary GBM tissues and adjacent tissues. Scale bar: original images–200 µ m, enlarged images–50 µ m. (**E**) ROC curve of IHC immunoreactivity scores of PD-L1 in 80 paraffin primary GBM tissues and 20 adjacent paraffin tissues. (**F**) Kaplan Meier survival analysis of PD-L1 in TCGA-GBM dataset. Left: Overall survival rate; Right: Disease free survival rate. *P < 0.05, ** *P* < 0.01, *** *P* < 0.001, **** *P* < 0.0001, ns: no significant.

Since PD-L1 has been reported to be highly expressed in GBM tissues and correlates with worse survival of patients with GBM^9,10^. We verified that PD-L1 RNA was significantly elevated in GBM when compared to normal corresponding tissues in TCGA-GBM dataset (Fig. 1A). Serum PD-L1 expression was greatly higher in GBM patients than that in healthy control (Fig. 1B). When compared to human astrocyte line HA1800, PD-L1 protein expression was remarkably upregulated in GBM cell lines T98 and U87 (Fig. 1C). IHC staining revealed that PD-L1 expression was elevated in GBM tissues relative to adjacent tissues (Fig. 1D). Additionally, area under receiver operating characteristic (ROC) curve (AUC) of IHC immunoreactivity score (IRS) of PD-L1 was 0.9234 (Fig. 1E, supplementary Fig. 1), indicating that PD-L1 RNA had high capacity in distinguishing healthy control and GBM. Moreover, GBM patients with high PD-L1 expression had worse overall survival and disease-free survival in TCGA-GBM dataset (Fig. 1F). Together, PD-L1 expression was upregulated in GBM and correlated with worse outcome of the patients with GBM.

### PD-L1 expression facilitated GBM cell viability, proliferation, migration and invasion

**Fig. 2:**
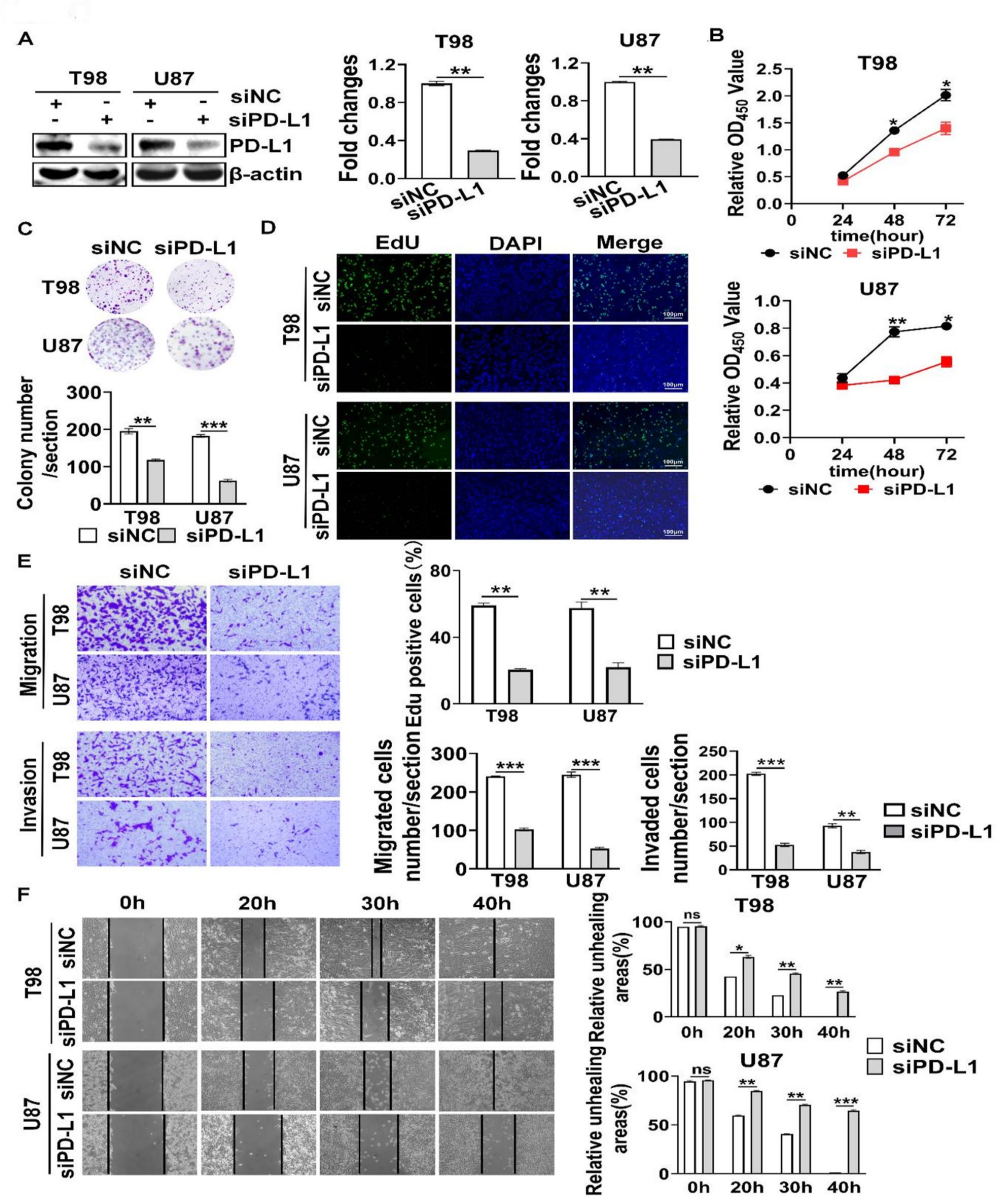
The effect of silencing PD-L1 on viability, proliferation, cell migration, invasion and scratch capacity of GBM cells. (**A**) Silencing PD-L1 in GBM cell lines. Western blot was used to detect PD-L1 protein expression. (**B**–**F**) Effect of silencing PD-L1 on GBM cell viability (**B**), colony formation (**C**), EdU incorporation (**D**), migration and invasion (**E**) and wound healing (**F**). * *P* < 0.05, ** *P* < 0.01, *** *P* < 0.001, ns: no significant.

To investigate the role of PD-L1 in GBM cell progression, PD-L1 was knockdown in GBM cells (Fig. 2A). Knockdown of PD-L1 expression decreased cell viability (Fig. 2B), cell proliferation (Fig. 2C, D). Since PD-L1 signaling has been show to regulate cell migration^19^, we examined whether PD-L1 mediated GBM cell migration and invasion. When compared to shNC, knockdown of PD-L1 expression decreased cell migration, cell invasion (Fig. 2E) and wound healing (Fig. 2F). Collectively, our results indicate that PD-L1 expression facilitated GBM cell viability, proliferation, migration and invasion.

### PD-L1 activated GP130/JAK2/STAT3 signaling pathway by interacting GP130

**Fig. 3:**
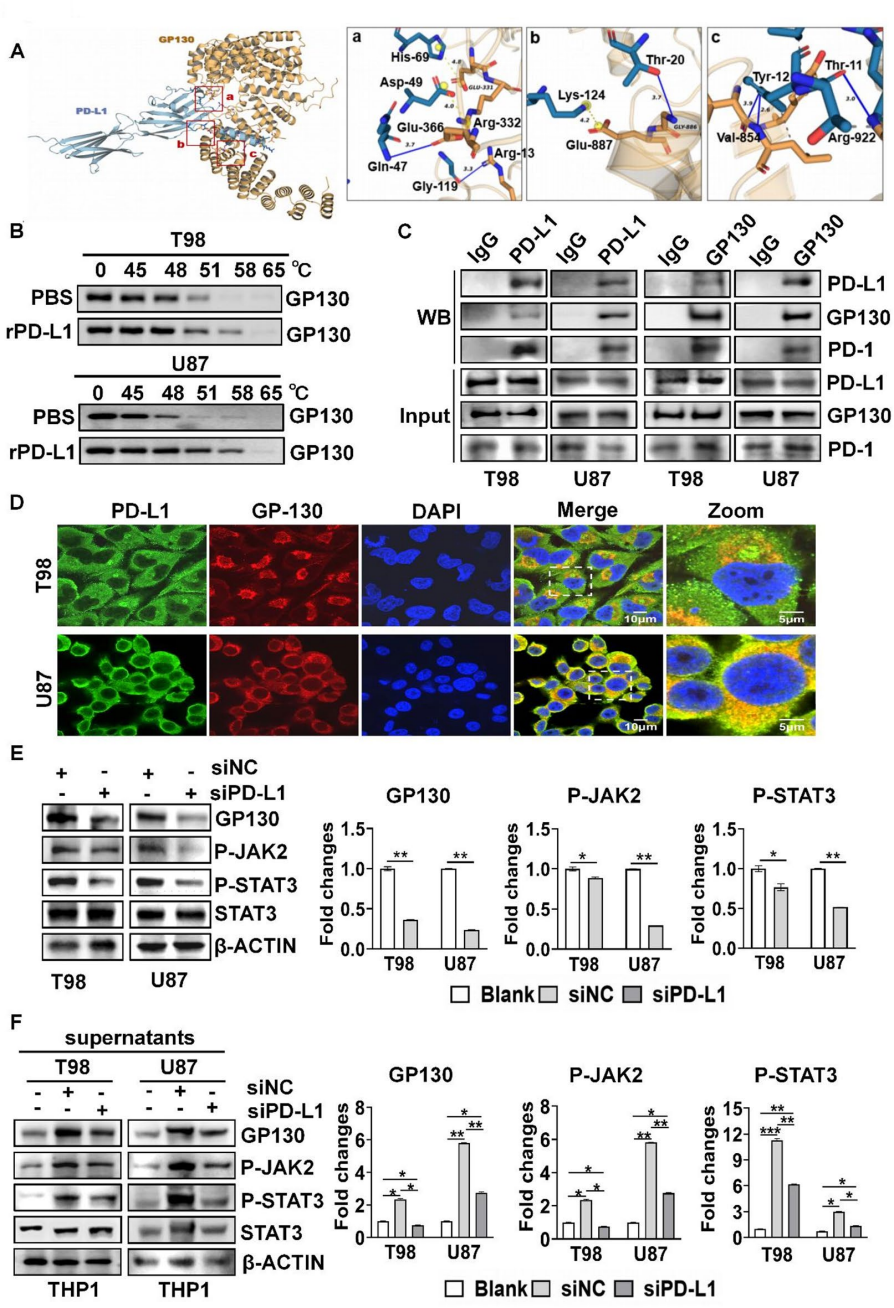
PD-L1 activated the GP130/JAK2/STAT3 signaling pathway by interacting with GP130. (**A**) Molecular docking analysis showing PD-L1 binding to GP130. (**B**) Effect of PD-L1 on GP130 protein stability. Cellular Thermal Shift Assay (CTSA) was used to determine GP130 - PD-L1 binding. (**C**) The interaction between PD-L1 and GP130 determined by Co-IP. (**D**) Co- localization of PD-L1 and GP130. (**E**) Effect of silencing PD-L1 on the protein expression levels of GP130, P-JAK2, P-STAT3 and STAT3 in GBM cells. (**F**) Effect of silencing PD-L1 in the supernatants derived GBM cell culture on the protein expression levels of GP130, P-JAK2, P-STAT3 and STAT3 in THP1 cells. * *P* < 0.05, ** *P* < 0.01, *** *P* < 0.001, ns: No statistically significant.

Correlation analysis revealed that PD-L1 was positively correlated with GP130, JAK2 and STAT3 in GBM (supplementary Fig. 2). Therefore, we performed molecular docking, revealing that PD-L1 interacted with GP130 with free energy of -45.21 (kcal/mol) (Fig. 3A). PD-L1 interacted with D2 and D3 domains of GP130 (Supplementary Fig. 3A, B) and these two domains are cytokine-binding module for activating GP130, suggesting that PD-L1 might activate GP130. CETSA showed that GP130 degradation was delayed upon recombinant PD-L1 (rPD-L1) treatment (Fig. 3B), indicating that PD-L1 did bind to GP130. To further determine whether PD-L1 interacted with GP130, we performed Co-IP experiment, showing that PD-L1 interacted with GP130 and PD-1 in GBM cells, and GP130 interacted with PD-L1 and PD-1 (Fig. 3C), indicating that PD-L1 interacted with both GP130 and its receptor PD-1. Immunofluorescent staining revealed that the partial colocalization between PD-L1 and GP130 was around cellular membranes, and the most of their co-localization was in cytoplasm in GBM cells (Fig. 3D). Since GP130 is involved in activating JAK2/STAT3 signaling pathway, we examined the effect of silencing PD-L1 on the activation GP130/JAK2/STAT3 signaling. Silencing PD-L1 significantly downregulated the expressions of GP130, phosphorylated JAK2 (P-JAK2) and phosphorylated STAT3 (P-STAT3) (Fig. 3E). In addition, the supernatants from GBM cells activated GP130/JAK2/STAT3 in THP1 cells, while silencing PD-L1 remarkably attenuated the activation of GP130/JAK2/STAT3 by the supernatants from GBM cells (Fig. 3F). These results indicate that PD-L1 activates GP130/JAK2/STAT3 pathway.

### PD-L1 promoted GBM cell viability, proliferation, migration and invasion capacity dependent on STAT3

**Fig. 4:**
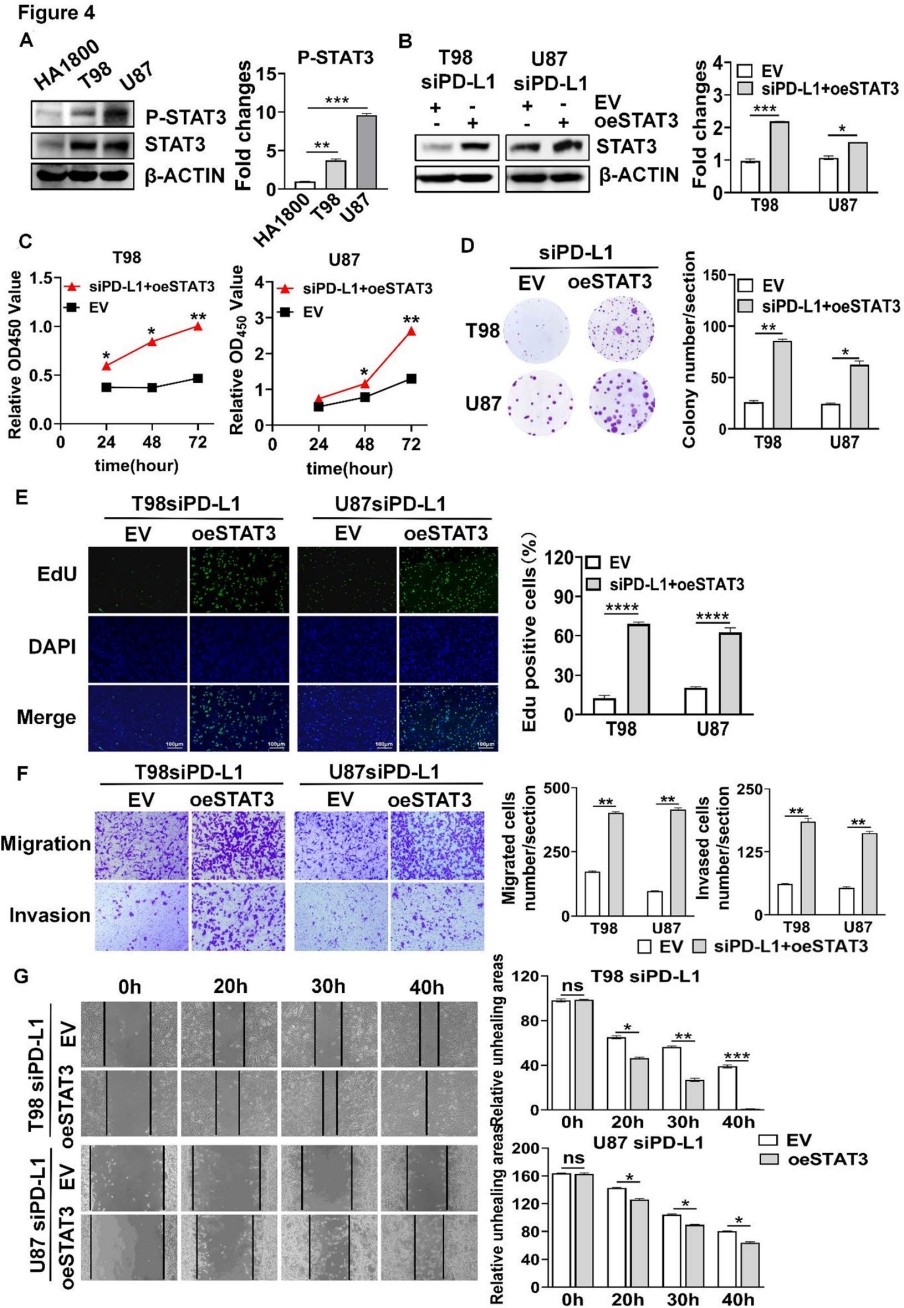
PD-L1 regulated cell viability, proliferation, migration, invasion capacity of GBM cells dependent on STAT3. (**A**) Protein expression levels of P-STAT3 and STAT3 in brain tissue and GBM cell lines. (**B**) Overexpression of STAT3 in GBM cells after silencing PD-L1. (**C**–**G**) Effect of overexpressing STAT3 on GBM cell viability (**C**), colony formation (**D**), EdU incorporation (**E**), migration and invasion (**F**) and wound healing (**G**). *P < 0.05, ** *P* < 0.01, *** *P* < 0.001, **** *P* < 0.0001, ns: No statistically significant.

To determine whether PD-L1 mediated GBM cell proliferation, migration and invasion via STAT3, we first examined whether STAT3 was activated in GBM cells. As shown in Fig. 4A, STAT3 was highly activated in GBM cells when compared to HA1800 cells. Next, we performed rescue experiment. After overexpressing STAT3 in GBM cells with knockdown of PD-L1 (Fig. 4B), cell viability, proliferation, migration and invasion as well as wound healing were restored (Fig. 4C-G), respectively. Collectively, these results indicate that PD-L1 mediates GBM cell viability, proliferation, migration, invasion capacity dependent on STAT3.

### STAT3 mediated IRAK2/NFκB/IL6 signaling pathway to promote GBM cell proliferation, migration and invasion

**Fig. 5:**
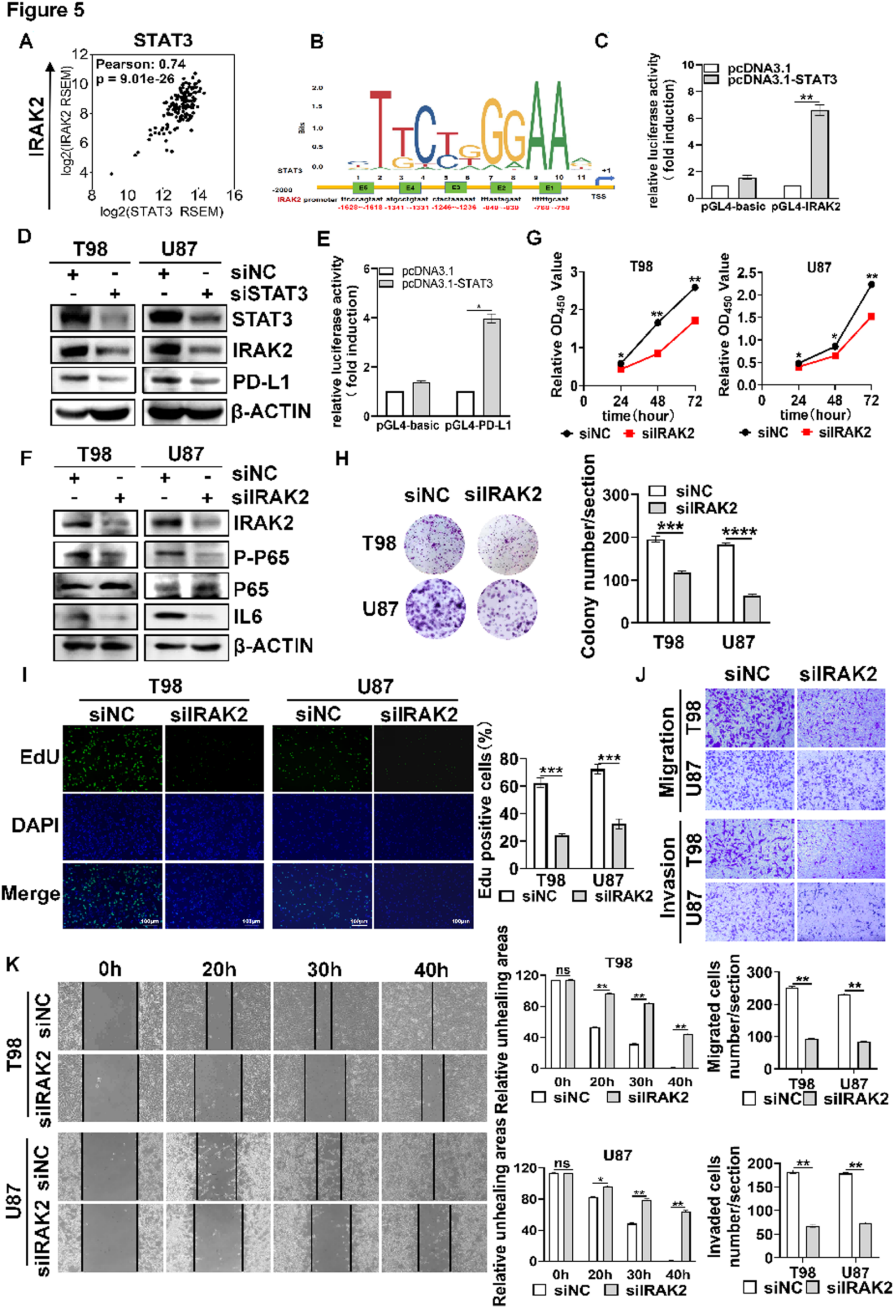
STAT3 regulated IRAK2/NFκB/IL6 signaling pathway. (**A**) Correlation of STAT3 expression and IRAK2 expression in TCGA-GBM dataset in cbioportal. (**B**) STAT3 binding of IRAK2 promoter. JASPAR was used to predict potential binding sites. (**C**) Dual luciferase reporter assay showing that STAT3 was bound to IRAK2 promoter. (**D**) Effect of silencing STAT3 on IRAK2 protein expression. Cells were transfected with siNC or siSTAT3. Protein expression levels of STAT3 and IRAK2 were detected by Western blot. (**E**) Dual luciferase reporter assay showing that STAT3 was bound to PD-L1 promoter. (**F**) Effect of silencing IRAK2 on P-P65, P65 and IL6 expressions. (**G**–**K**) Effect of silencing IRAK2 on cell viability (**G**), colony formation (**H**), proliferation (**I**), migration and invasion (**J**) as well as wound healing capacity (**K**). *P < 0.05, ** *P* < 0.01, *** *P* < 0.001, **** *P* < 0.0001, ns: No statistically significant.

STAT3 has multiple targets as a transcription factor. To screen STAT3 targets in GBM, we looked for the targets of STAT3 which were highly positively correlated with STAT3 in GBM through TCGA-GBM dataset in cbioportal. We found that IRAK2 was positively correlated with STAT3 (Fig. 5A, Pearson *R* = 0.74, *P* = 9.01e-26). STAT3 has 5 potential binding sites in IRAK2 promoter (Fig. 5B). E5 box contains TTCN_2 − 4_GAA, which is gamma-activated sequence (GAS) element specific for STAT transcription factors^20^, indicating that IRAK2 is a potential target of STAT3. If IRAK2 is a target of STAT3, IRAK2 was supposed to be upregulated in GBM cells. Our result verified that IRAK2 was remarkably elevated in GBM cells relative to HA1800 cells (Supplementary Fig. 4). Dual luciferase reporter assay showed that STAT3 transcription activity was upregulated after U87 cells were co-transfected with IRAK2 promoter and STAT3 when compared to pGL4-basic (Fig. 5C). Therefore, we further examined whether STAT3 mediated IRAK2 expression. Silencing STAT3 downregulated IRAK2 expression (Fig. 5D). Since STAT3 was also shown to be an upstream of PD-L1 in lung cancer^21^, we examined whether STAT3 regulated PD-L1 expression in GBM. As expected, PD-L1 expression was decreased upon silencing STAT3 (Fig. 5D). We further investigated whether STAT3 transcriptionally mediated PD-L1 expression. STAT3 is shown to have potential binding sites on PD-L1 promoter (Supplementary Fig. 5A). Additionally, the ChIP peaks of active histone marks (H3K4me3, H3K27Ac) were enriched on PD-L1 promoter in U87 cells (Supplementary 5B), indicating that PD-L1 promoter is active for transcription. STAT3 ChIP peaks were also enriched on PD-L1 promoter in several cell lines (Supplementary Fig. 5B), suggesting that STAT3 is able to bind to PD-L1 promoter. To validate whether STAT3 bound to PD-L1 promoter, we conducted dual luciferase reporter assay. As illustrated in Fig. 5E, STAT3 transcription activity was upregulated after U87 cells were co-transfected with PD-L1 promoter and STAT3 when compared to pGL4-basic, indicating that STAT3 is able to bind PD-L1 promoter.

Since IRAK2 is known to activate NFκB transcription factor, which transcripts IL6 expression, we first examined whether IRAK2 expression was correlated with interleukin-6 receptor (IL6R) and IL6 in GBM. We found that IRAK2 was positively correlated with IL6R and IL6 (Supplementary Fig. 6). Silencing IRAK2 inhibited NF-κB p65 (P65) activity and downregulated IL6 (Fig. 5F), GBM cell viability (Fig. 5G), colony formation (Fig. 5H), proliferation (Fig. 5I), cell migration and invasion (Fig. 5J) and wound healing capacity (Fig. 5K). These results indicate that STAT3 regulate IRAK2/NFκB/IL6 signaling pathway to facilitate GBM cell proliferation, migration and invasion.

### STAT3 promoted GBM cell viability, proliferation, migration, invasion and wound healing capacity via IRAK2

**Fig. 6:**
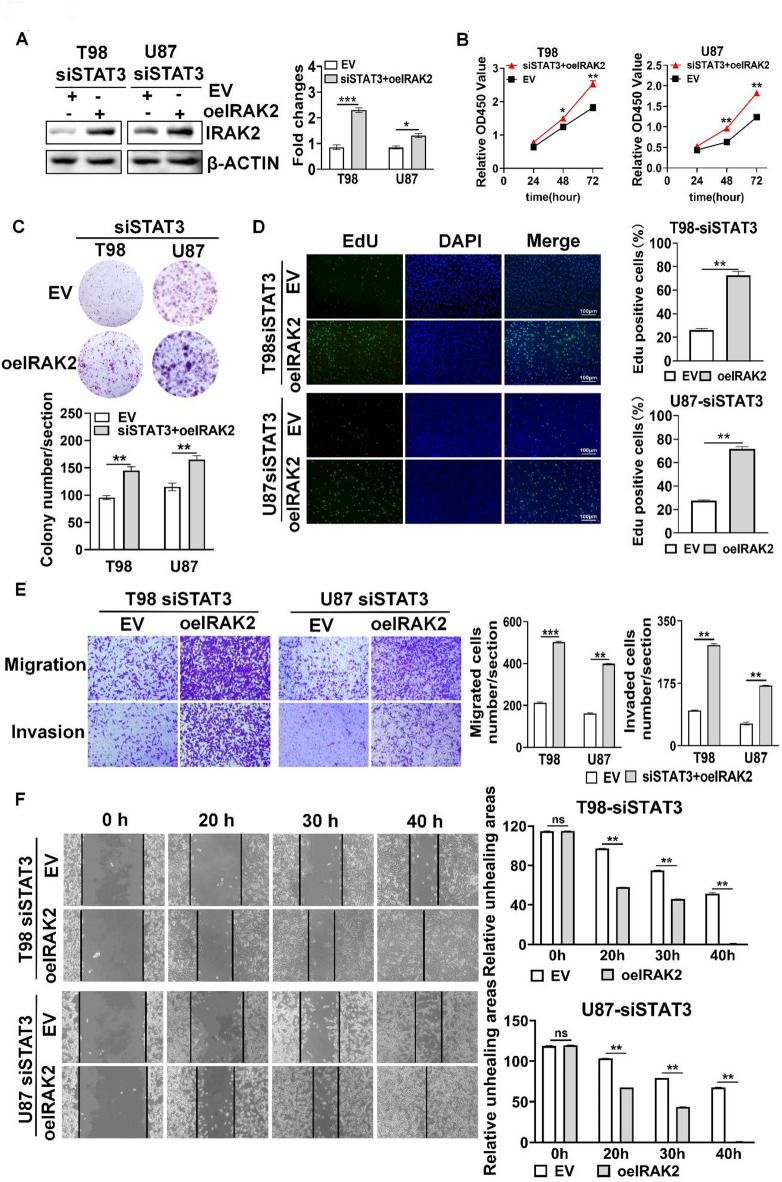
STAT3 promoted GBM cell viability, proliferation, migration, invasion and wound healing capacity via IRAK2. (**A**) Overexpression of IRAK2 in GBM cells after silencing STAT3. (**B**–**F**) Effect of overexpressing IRAK2 on GBM cell viability (**B**), colony formation (**C**), EdU incorporation (**D**), migration and invasion (**E**) and wound healing (**F**). *P < 0.05, ** *P* < 0.01, *** *P* < 0.001, ns: No statistically significant.

To determine whether STAT3 depended on IRAK2 to mediate GBM cell viability, proliferation, migration, invasion and wound healing capacity, we performed rescue experiments. Overexpressing IRAK2 in GBM cells with STAT3 silencing restored IRAK2 expression (Fig. 6A), GBM cell viability (Fig. 6B), colony formation (Fig. 6C), proliferation (Fig. 6D), cell migration and invasion (Fig. 6E) and wound healing capacity (Fig. 6F). Collectively, these results indicate that STAT3 regulate facilitate GBM cell proliferation, migration and invasion dependent on IRAK2 expression.

### PD-L1-regulated IL6 was associated with Myeloid-derived suppressor cell (MDSC), GBM-associated macrophage and inhibition of dendritic cells

**Fig. 7:**
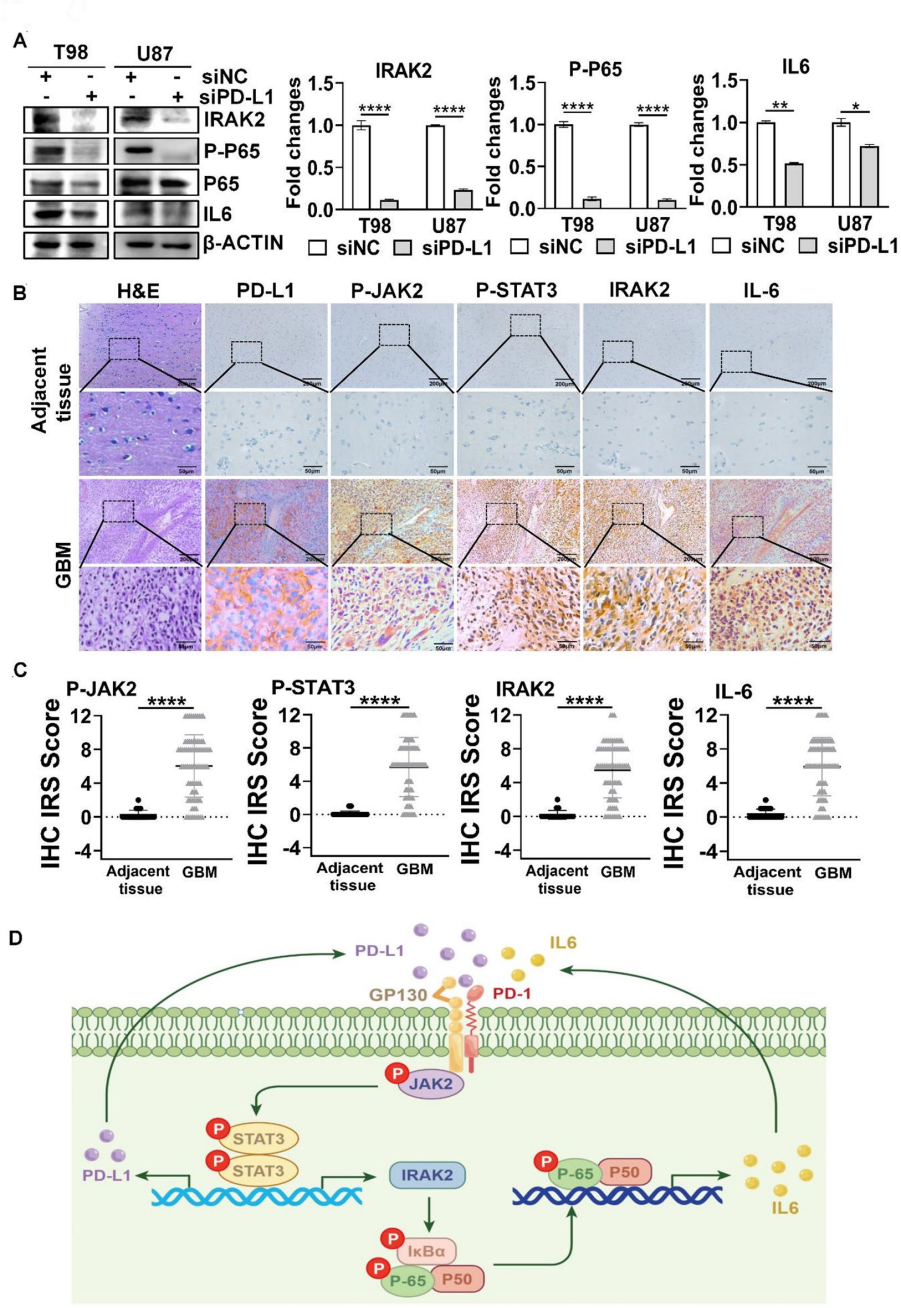
Correlation between PD-L1 and P-JAK2, P-STAT3, IRAK2 and IL6. (**A**) Effect of silencing PD-L1 on the expression levels of indicated proteins in GBM cells. (**B**) Representative IHC images of P-JAK2, P-STAT3, IRAK2, and IL6 proteins in human GBM tissues. Scale bar: original images --200 μm and the enlarged images–50 μm. (**C**) IHC scores of P-JAK2, P-STAT3, IRAK2, and IL6. *P < 0.05, **P < 0.01, ****P < 0.0001. (**D**) Proposed model for PD-L1-mediated GBM cell proliferation, migration and invasion.

Since we showed that PD-L1 activated GP130/JAK2/STAT3 and STAT3 regulated IRAK2 expression, we examined whether PD-L1 may regulate IRAK2/NFκB/IL6. Silencing PD-L1 greatly downregulated IRAK2, phosphorylated NF-κB p65 (P-P65) and IL6 expression (Fig. 7A). To determine the clinical association of PD-L1 expression with the expressions of P-JAK2, P-STAT3, IRAK2 and IL6, we performed H&E staining and IHC staining. H&E staining results demonstrated that the clinical samples were correct. IHC staining showed that PD-L1 was highly expressed in human GBM tissues, accompanied by elevated expressions of P-JAK2, P-STAT3, IRAK2 and IL6 (Fig. 7B), supporting by their IHC scores (Fig. 7C). The correlation analysis of IHC scores of these proteins revealed that these molecules were positively correlated with each other (Supplementary Fig. 7). Together, our results indicate that PD-L1 mediate JAK2/STAT3/IRAK2/NFκB/IL6 signaling pathway.

Our bioinformatic analysis showed that IL6 RNA expression was positively not only with the RNA expression levels of macrophage-associated genes CXCL1, CXCL2, CXCL12, CCL2, CCL20, TGFβ(supplementary Fig. 8A), which are known to promote MDSC expansion and function in GBM^22^, but also with the RNA expression levels of IL10, EGF, TGFβ, LOX and SPP1 as well as macrophage markers (supplementary Fig. 8B) which have been shown to promote macrophage infiltration in GBM^22,23^. Moreover, IL6 was positively with the RNA expression levels of S100A8, S100A9, CXCL1 and CXCL8 (supplementary Fig. 8C), which have been reported to inhibit dendritic cells in GBM^22^, suggesting that PD-L1 mediated IL6 is associated with immunosuppression in GBM via involving in MDSC, GBM-associated macrophage and inhibition of dendritic cells. Based on our results, we proposed a model (Fig. 7D), in which PD-L1 promotes GBM cell proliferation, migration and invasion via its autoregulation by activating GP130/JAK2/STAT3/NFκB/IRAK2/IL6 pathway.

## Discussion

PD-L1 confers to GBM refractory to therapy. In this study, we demonstrate that PD-L1 facilitates in GBM cell proliferation, migration and invasion through activating GP130/JAK2/STAT3/IRAK2 /NFκB/IL6 pathway. We found that PD-L1 activate GP130 via interacting GP130/JAK2/STAT3 signaling. STAT3 transcriptionally promotes IRAK2 and PD-L1 expressions. IRAK2 promotes IL6 expression via activating NFκB signaling. This study demonstrates that PD-L1 and IL6 persistently mediate GBM progression via forming several feedback loops, highlighting that PD-L1 ~ IL6 loop blockade may be a promising strategy for GBM therapy.

Our finding showed that PD-L1 facilitates GBM cell proliferation, migration and invasion via interacting GP130/JAK2/STAT3.We first demonstrated that PD-L1 interacted with GP130 and PD-1. Our study supports that PD-L1 activates JAK2/STAT3 in non-small-cell lung cancer cells^24^, but the mechanism by which PD-L1 activated JAK2/STAT3 is different. PD-L1 has shown to bind and inhibit protein tyrosine phosphatase 1B, and also bind to p-Stat3 in the nucleus, thus promoting the activity of p-STAT3^24^, while our results showed that PD-L1 interacted with D2 and D3 domains of GP130 and these domains are indispensable for GP130 activation^25^. CESTA also showed that GP130 degradation was reduced upon PD-L1 treatment, indicating that the interaction between PD-L1 and GP130 stabilizes GP130. Co-IP experiments demonstrated that PD-L1 interacted with GP130 and PD-1, and GP130 interacted with PD-L1 and PD-1. Additionally, we found that the partial of PD-L1 ~ GP130 interaction was localized around cellular membrane, but the most of the interaction occurred in cytoplasm, consistent with the study showing that constitutively active GP130 variants exhibit a reduced expression level on cell surface^25^, leading to the abundant GP130 in cytoplasm^25^. It is plausible that the most of PD-L1 ~ GP130 interaction existed in the cytoplasm. These results suggest that PD-L1 might activate GP130 via interacting with PD-1/GP30 complex, leading to activate JAK2/STAT3.

Our further results demonstrate that PD-L1 facilitates GBM cell proliferation, migration and invasion dependent on STAT3. Overexpressing STAT3 restored silencing PD-L1-mediated inhibition of GBM cell proliferation, migration and invasion. We show that STAT3 promoted GBM cell proliferation, migration and invasion via STAT3, consistent with that STAT3 promotes GBM progression^26,27^. These results demonstrate that PD-L1 facilitates GBM cell proliferation, migration and invasion via STAT3.

Our finding revealed that STAT3 facilitates GBM cell proliferation, migration and invasion dependent on IRAK2 signaling. The link between IRAKs and STAT3 is barely reported. IRAK-M has been shown to stabilize STAT3 protein in colorectal cancer progression^28^. We showed that IRAK2 promoter has GAS element for STAT protein binding, and dual luciferase reporter assay confirmed that STAT3 is capable of binding IRAK2 promoter. Silencing STAT3 downregulated IRAK2 expression. Silencing IRAK2 reduced GBM cell proliferation, migration and invasion. Overexpressing IRAK2 reversed STAT3 inhibition-mediated effect on GBM cell proliferation, migration and invasion, supporting that STAT3 depends on IRAK2 to promote GBM cell proliferation, migration and invasion.

Our results support that PD-L1 and IL6 persistently induce immunosuppressive GBM. First, PD-L1 and IL-6 are constitutively and highly expressed in GBM. PD-L1 expression has been promoted by multiple activation pathways such as Toll-like receptors (TLR), epidermal growth factor (EGFR), interferon alpha receptor (IFNAR) and interferon-gamma receptor (IFNGR)^29^. Our finding demonstrated that PD-L1 expression can be autoregulated. PD-L1 autoregulates its expression via two loops: (1) PD-L1/GP130/JAK2/STAT3/PD-L1 and (2) PD-L1/GP130/JAK2/STAT3/IRAK2/NFκB/IL-6/GP130/STAT3/PD-L1.Therefore, it is plausible that PD-L1 and IL-6 are constitutively and highly expressed in GBM. Second, both PD-L1 and IL6 activate GP130/JAK2/STAT3 pathway. STAT3 is reported to promote PD-L1 expression in GBM cells^12^, and our results showed that STAT3 transcriptionally promote PD-L1 expression. GBM-derived IL-6 have been shown to facilitate peripheral immunosuppressive myeloid cell PD-L1 expression in STAT3-dependent manner in GBM^17^. Additionally, STAT3 is a master to promote GBM suppression. Therefore, these pathways confer to PD-L1 autoregulation. Third, our bioinformatic analysis reveals that PD-L1 mediated IL6 is positively associated with MDSC, GBM-associated macrophage and inhibition of dendritic cells, which are known to create immunosuppression in GBM, consistent with GBM-derived IL6 mediates immunosuppressive peripheral myeloid cells^17^. IL6 is a key immunomodulatory cytokine that promotes tumor survival, growth and induce immunosuppressive microenvironment through classic signaling, trans-signaling and cluster signaling^30^, and IL6 signaling in CD8^+^ T promotes resistance to anti-PD-L1 immunotherapy^31^. Additionally, IL6 is a target for GBM immunotherapy^32^. Given that both PD-L1 and IL6 can trigger STAT3 activity which is a central hub in promoting immunosuppression in GBM^12,33^, our results suggest that bio-specific anti-IL6xPD-L1 is an option that targeting PD-L1/PD-1 signals for GBM therapy.

There are several limitations to this study. First, we did not clarify the role PD-L1-GP130 interaction in cytoplasm. Second, we did not examine immunosuppression-related markers in primary GBM samples. Third, we did not examine the effect of simultaneous targeting PD-L1 and IL6 on GBM immune microenvironment in vivo. In spite of these limitations, we demonstrate that PD-L1/GP130/JAK2/STAT3/IRAK2/IL6 is clinically relevant to GBM. Our study suggests that dual targeting PD-L1 and IL6 might be a promising target for GBM therapy.

## Conclusions

Our results indicate that PD-L1 promotes GBM cell viability, proliferation, migration and invasion via interacting with GP130 & PD-1 and activating JAK2/STAT3/IRAK2/NFκB/IL6 axis in GBM cells. Additionally, STAT3 transcriptionally promotes PD-L1 expression, thus our results demonstrate that PD-L1 may constitutively promote its expression, IL6 expression and STAT3 activation, which confers to GBM suppression.

## Supplementary Information

Below is the link to the electronic supplementary material.

Supplementary Material 1

Supplementary Material 2

Supplementary Material 3

## Data Availability

The data that support the findings of this study are available from the corresponding author upon reasonable request.

## References

[1] Longhitano, L. et al. Lactate modulates microglia polarization via IGFBP6 expression and remodels tumor microenvironment in glioblastoma. *Cancer Immunol. Immunother*. **72**, 1–20 (2023). 35654889 10.1007/s00262-022-03215-3 PMC9813126

[2] Longhitano, L. et al. Li volti, lactate induces the expressions of MCT1 and HCAR1 to promote tumor growth and progression in glioblastoma. *Front. Oncol.* **12**, 871798 (2022). 35574309 10.3389/fonc.2022.871798 PMC9097945

[3] Medikonda, R., Dunn, G., Rahman, M., Fecci, P. & Lim, M. A review of glioblastoma immunotherapy. *J. Neurooncol*. **151**, 41–53 (2021). 32253714 10.1007/s11060-020-03448-1

[4] Jackson, C. M., Choi, J. & Lim, M. Mechanisms of immunotherapy resistance: lessons from glioblastoma. *Nat. Immunol.* **20**, 1100–1109 (2019). 31358997 10.1038/s41590-019-0433-y

[5] Yang, T., Kong, Z. & Ma, W. PD-1/PD-L1 immune checkpoint inhibitors in glioblastoma: clinical studies, challenges and potential. *Hum. Vaccin Immunother*. **17**, 546–553 (2021). 32643507 10.1080/21645515.2020.1782692 PMC7899692

[6] de Groot, J. et al. Window-of-opportunity clinical trial of pembrolizumab in patients with recurrent glioblastoma reveals predominance of immune-suppressive macrophages. *Neuro Oncol.* **22**, 539–549 (2020). 31755915 10.1093/neuonc/noz185 PMC7158647

[7] Lim, M., Xia, Y., Bettegowda, C. & Weller, M. Current state of immunotherapy for glioblastoma. *Nat. Rev. Clin. Oncol.* **15**, 422–442 (2018). 29643471 10.1038/s41571-018-0003-5

[8] Chryplewicz, A. et al. Cancer cell autophagy, reprogrammed macrophages, and remodeled vasculature in glioblastoma triggers tumor immunity. *Cancer Cell.* **40**, 1111–1127e1119 (2022). 36113478 10.1016/j.ccell.2022.08.014 PMC9580613

[9] Wang, Z. et al. Molecular and clinical characterization of PD-L1 expression at transcriptional level via 976 samples of brain glioma. *Oncoimmunology*. **5**, e1196310 (2016). 10.1080/2162402X.2016.1196310 PMC5139638 27999734

[10] Nduom, E. K. et al. PD-L1 expression and prognostic impact in glioblastoma. *Neuro Oncol.* **18**, 195–205 (2016). 26323609 10.1093/neuonc/nov172 PMC4724183

[11] Wang, Q. W. et al. MET overexpression contributes to STAT4-PD-L1 signaling activation associated with tumor-associated, macrophages-mediated immunosuppression in primary glioblastomas. *J. Immunother Cancer*. **9** (2021). 10.1136/jitc-2021-002451 PMC8527154 34667077

[12] Tong, L. et al. ACT001 reduces the expression of PD-L1 by inhibiting the phosphorylation of STAT3 in glioblastoma. *Theranostics* **10**, 5943–5956 (2020). 32483429 10.7150/thno.41498 PMC7254983

[13] Pang, K. et al. Research progress of therapeutic effects and drug resistance of immunotherapy based on PD-1/PD-L1 Blockade. *Drug Resist. Updat*. **66**, 100907 (2023). 36527888 10.1016/j.drup.2022.100907

[14] Hegde, P. S., Wallin, J. J. & Mancao, C. Predictive markers of anti-VEGF and emerging role of angiogenesis inhibitors as immunotherapeutics. *Semin Cancer Biol.* **52**, 117–124 (2018). 29229461 10.1016/j.semcancer.2017.12.002

[15] Frentzas, S. et al. Phase 1a dose escalation study of Ivonescimab (AK112/SMT112), an anti-PD-1/VEGF-A bispecific antibody, in patients with advanced solid tumors. *J. Immunother Cancer*, **12** (2024). 10.1136/jitc-2023-008037 PMC11033648 38642937

[16] Tang, Z. et al. GEPIA: a web server for cancer and normal gene expression profiling and interactive analyses. *Nucleic Acids Res.* **45**, W98–w102 (2017). 28407145 10.1093/nar/gkx247 PMC5570223

[17] Lamano, J. B. et al. Glioblastoma-derived IL6 induces immunosuppressive peripheral myeloid cell PD-L1 and promotes tumor growth. *Clin. Cancer Res.* **25**, 3643–3657 (2019). 30824583 10.1158/1078-0432.CCR-18-2402 PMC6571046

[18] Zhang, Q. et al. Serum-resistant CpG-STAT3 decoy for targeting survival and immune checkpoint signaling in acute myeloid leukemia. *Blood* **127**, 1687–1700 (2016). 26796361 10.1182/blood-2015-08-665604 PMC4817311

[19] Piao, W. et al. PD-L1 signaling selectively regulates T cell lymphatic transendothelial migration. *Nat. Commun.* **13**, 2176 (2022). 35449134 10.1038/s41467-022-29930-0 PMC9023578

[20] Decker, T., Kovarik, P. & Meinke, A. GAS elements: a few nucleotides with a major impact on cytokine-induced gene expression. *J. Interferon Cytokine Res.* **17**, 121–134 (1997). 9085936 10.1089/jir.1997.17.121

[21] Xie, C. et al. Apatinib triggers autophagic and apoptotic cell death via VEGFR2/STAT3/PD-L1 and ROS/Nrf2/p62 signaling in lung cancer. *J. Exp. Clin. Cancer Res.* **40**, 266 (2021). 34429133 10.1186/s13046-021-02069-4 PMC8385858

[22] Lin, H. et al. Understanding the immunosuppressive microenvironment of glioma: mechanistic insights and clinical perspectives. *J. Hematol. Oncol.* **17**, 31 (2024). 38720342 10.1186/s13045-024-01544-7 PMC11077829

[23] Chen, P. et al. Symbiotic Macrophage-Glioma cell interactions reveal synthetic lethality in PTEN-Null glioma. *Cancer Cell.* **35**, 868–884e866 (2019). 31185211 10.1016/j.ccell.2019.05.003 PMC6561349

[24] Jeong, H. et al. Cell-intrinsic PD-L1 signaling drives immunosuppression by myeloid-derived suppressor cells through IL-6/Jak/Stat3 in PD-L1-high lung cancer. *J. Immunother Cancer*. **13** (2025). 10.1136/jitc-2024-010612 PMC11887297 40050048

[25] Lamertz, L., Floss, D. M. & Scheller, J. Combined deletion of the fibronectin-type III domains and the stalk region results in ligand-independent, constitutive activation of the Interleukin 6 signal-transducing receptor gp130. *Cytokine* **110**, 428–434 (2018). 29789201 10.1016/j.cyto.2018.05.011

[26] Gao, X. et al. Circular RNA-encoded oncogenic E-cadherin variant promotes glioblastoma tumorigenicity through activation of EGFR-STAT3 signalling. *Nat. Cell. Biol.* **23**, 278–291 (2021). 33664496 10.1038/s41556-021-00639-4

[27] Li, H. et al. miR-519a enhances chemosensitivity and promotes autophagy in glioblastoma by targeting STAT3/Bcl2 signaling pathway. *J. Hematol. Oncol.* **11**, 70 (2018). 29843746 10.1186/s13045-018-0618-0 PMC5975545

[28] Kesselring, R. et al. Fichtner-Feigl, IRAK-M expression in tumor cells supports colorectal cancer progression through reduction of antimicrobial defense and stabilization of STAT3. *Cancer Cell.* **29**, 684–696 (2016). 27150039 10.1016/j.ccell.2016.03.014

[29] Litak, J., Mazurek, M., Grochowski, C., Kamieniak, P. & Roliński, J. PD-L1/PD-1 axis in glioblastoma multiforme. *Int. J. Mol. Sci.*, **20** (2019). 10.3390/ijms20215347 PMC6862444 31661771

[30] Rose-John, S., Jenkins, B. J., Garbers, C., Moll, J. M. & Scheller, J. Targeting IL-6 trans-signalling: past, present and future prospects. *Nat. Rev. Immunol.* **23**, 666–681 (2023). 37069261 10.1038/s41577-023-00856-y PMC10108826

[31] Huseni, M. A. et al. CD8(+) T cell-intrinsic IL-6 signaling promotes resistance to anti-PD-L1 immunotherapy. *Cell. Rep. Med.* **4**, 100878 (2023). 36599350 10.1016/j.xcrm.2022.100878 PMC9873827

[32] Yang, F. et al. Synergistic immunotherapy of glioblastoma by dual targeting of IL-6 and CD40. *Nat. Commun.* **12**, 3424 (2021). 34103524 10.1038/s41467-021-23832-3 PMC8187342

[33] Hussain, S. F. et al. A novel small molecule inhibitor of signal transducers and activators of transcription 3 reverses immune tolerance in malignant glioma patients. *Cancer Res.* **67**, 9630–9636 (2007). 17942891 10.1158/0008-5472.CAN-07-1243

