# Supplementary material for: PD-L1 autoregulation promotes the proliferation, migration and invasion of glioblastoma cells via GP130/JAK2/STAT3/IRAK2/IL6 signaling pathway
Source: Sci Rep. 2025 Oct 8;15:35186. doi: 10.1038/s41598-025-19169-2 (PMC12508128; doi:10.1038/s41598-025-19169-2)
Supplement: Supplementary file 1 — Supplementary Material 1 [file 41598_2025_19169_MOESM1_ESM.docx]

| **Supplementary Table 1 Expressions of PD-L1 in human GBM and Adjacent tissues** | | | | | |
| --- | --- | --- | --- | --- | --- |
|  | | | | | |
| **Group** | **NO. of Patients** | **PD-L1 expression** | | | |
|  |  | **negative** | **positive** | **χ2** | **P Value** |
| Adjacent tissue | 20 | 16 | 4 | 39.07^a^ | P＜0.0001 |
| GBM | 80 | 10 | 70 |  |  |
| a: Continuous Correction | | | | |  |

| **Supplementary Table 2 Antibodies used in this study** | | | | |
| --- | --- | --- | --- | --- |
| **antibody** | **manufacturers** |  | **Cat No.** |  |
| anti-PD-L1 | Proteintech | Rabbit PolyAb | 17952-1-AP |  |
| anti-GP130（LRPPRC） | Proteintech | Rabbit PolyAb | 21175-1-AP |  |
| anti-Phospho-JAK2 | abmart | Rabbit MonoclonalAb | T56570 |  |
| anti-JAK2 | CST | Rabbit MonoclonalAb | 3230S |  |
| anti-phospho-Tyr705-STAT3 | CST | Rabbit MonoclonalAb | 9145S |  |
| anti-STAT3 | Proteintech | Rabbit PolyAb | 10253-2-AP |  |
| anti-IRAK2 | Affinity | Rabbit MonoclonalAb | DF4782 |  |
| anti-P65 | Wanlei | Rabbit PolyAb | WL01273b |  |
| anti-P-P65 | abmart | Rabbit PolyAb | TP56372 |  |
| anti-IL6 | Proteintech | Rabbit PolyAb | 26404-1-AP |  |
| anti-β-ACTIN | Proteintech | Rabbit MonoclonalAb | 66009-1-Ig |  |

**Supplementary Table 3 siRNA sequences**

| **Gene name** | **Sequences** | |
| --- | --- | --- |
|  | sense（5'-3'） | antisense（5'-3'） |
| PD-L1 | GACCUAUAUGUGGUAGAGUAU | AUACUCUACCACAUAUAGGUC |
| IRAK2 | GACUUACUCCUCAGUGAUAUU | AAUAUCACUGAGGAGUAAGUC |
| stat3 | GGAAGCUGCAGAAAGAUACGACUGA | UCAGUCGUAUCUUUCUGCAGCUUCC |
