# Supplementary material for: PD-L1 autoregulation promotes the proliferation, migration and invasion of glioblastoma cells via GP130/JAK2/STAT3/IRAK2/IL6 signaling pathway
Source: Sci Rep. 2025 Oct 8;15:35186. doi: 10.1038/s41598-025-19169-2 (PMC12508128; doi:10.1038/s41598-025-19169-2)

Fig.1C

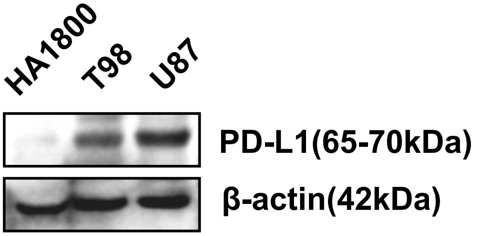

HA1800
T98
U87
PD-L1(65-70kDa)
β-actin(42kDa)

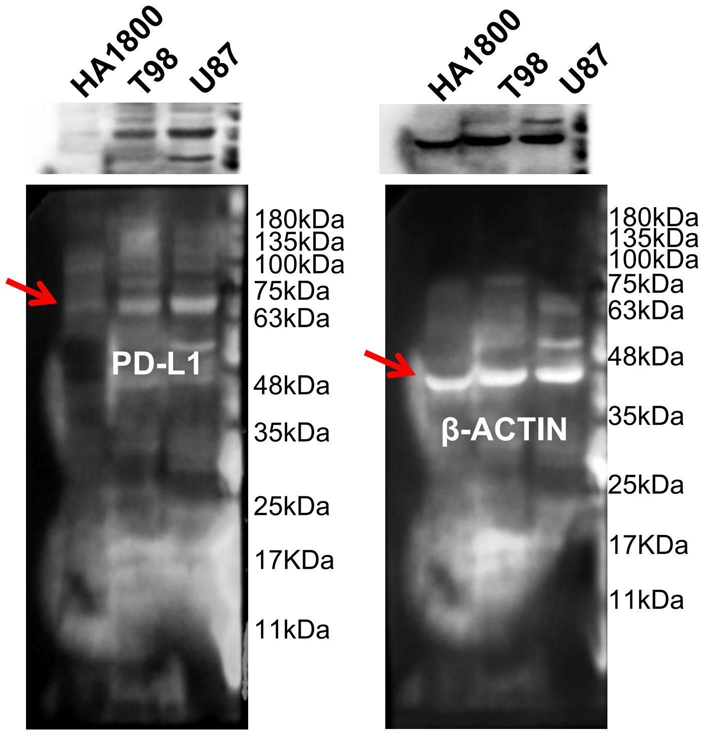

HA1800
T98
U87
PD-L1
180kDa
135kDa
100kDa
75kDa
63kDa
48kDa
35kDa
25kDa
17KDa
11kDa
HA1800
T98
U87
β-ACTIN
180kDa
135kDa
100kDa
75kDa
63kDa
48kDa
35kDa
25kDa
17KDa
11kDa

Fig.2A

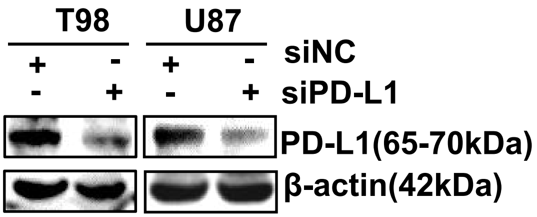

T98
U87
siNC
siPD-L1
+ - + -
- + - +
PD-L1(65-70kDa)
β-actin(42kDa)

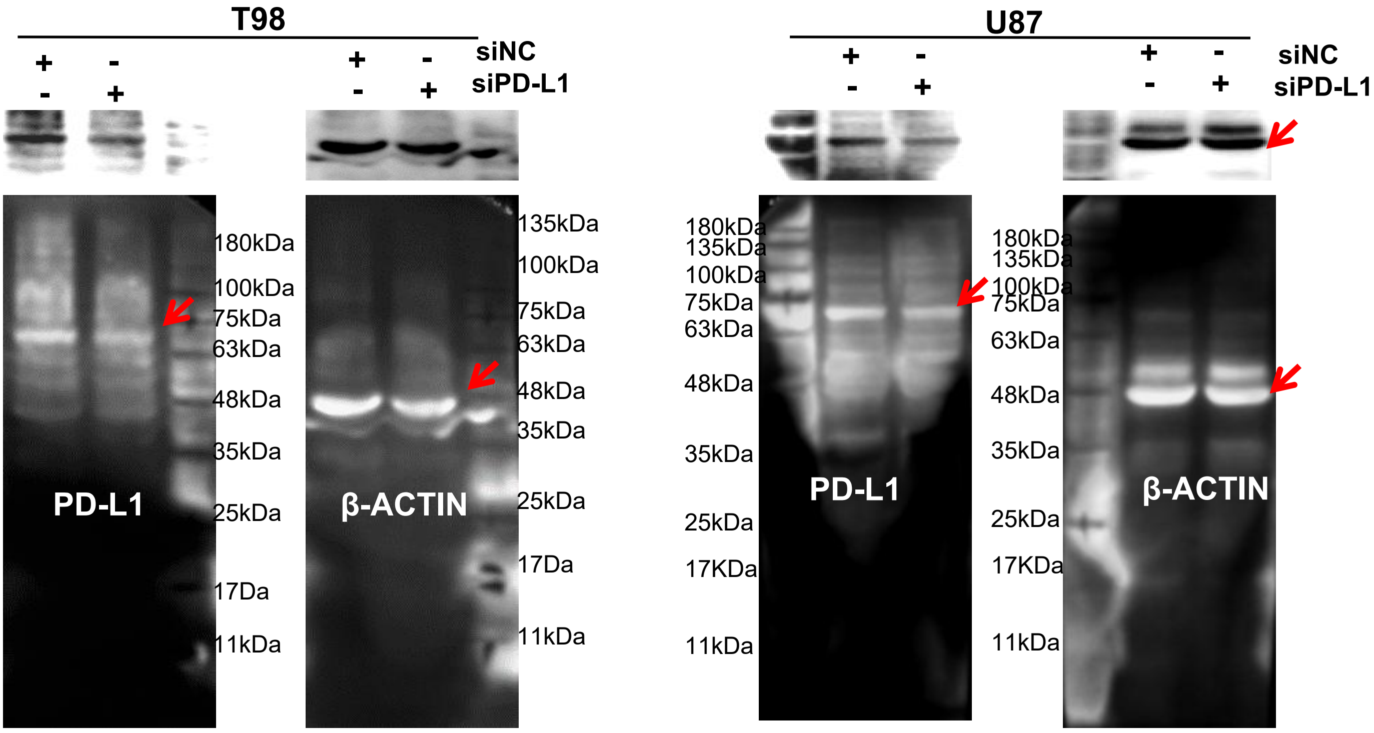

T98
siNC
siPD-L1
+ -
- +
PD-L1
180kDa
100kDa
75kDa
63kDa
48kDa
35kDa
25kDa
17Da
11kDa
β-ACTIN
135kDa
100kDa
75kDa
63kDa
48kDa
35kDa
25kDa
17Da
11kDa
U87
siNC
siPD-L1
PD-L1
180kDa
135kDa
100kDa
75kDa
63kDa
48kDa
35kDa
25kDa
17KDa
11kDa
β-ACTIN
180kDa
135kDa
100kDa
75kDa
63kDa
48kDa
35kDa
25kDa
17KDa
11kDa

Fig.3B

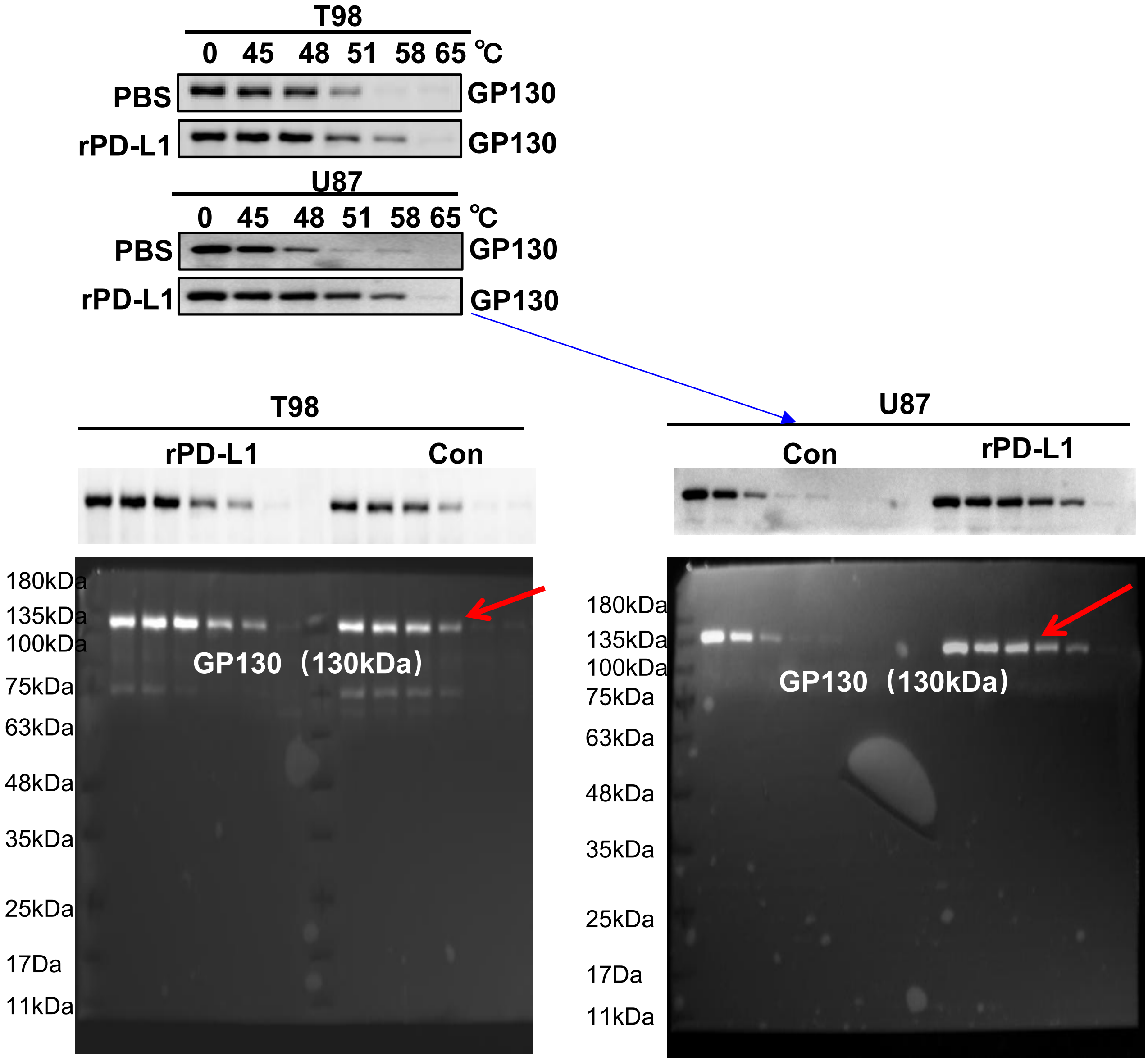
T98
0 45 48 51 58 65 ℃
PBS
GP130
rPD-L1
GP130
U87
0 45 48 51 58 65 ℃
PBS
GP130
rPD-L1
GP130
T98
rPD-L1
Con
180kDa
135kDa
100kDa
75kDa
63kDa
48kDa
35kDa
25kDa
17Da
11kDa
GP130 （130kDa）
U87
Con
rPD-L1
180kDa
135kDa
100kDa
75kDa
63kDa
48kDa
35kDa
25kDa
17Da
11kDa
GP130 （130kDa）

Fig.3C

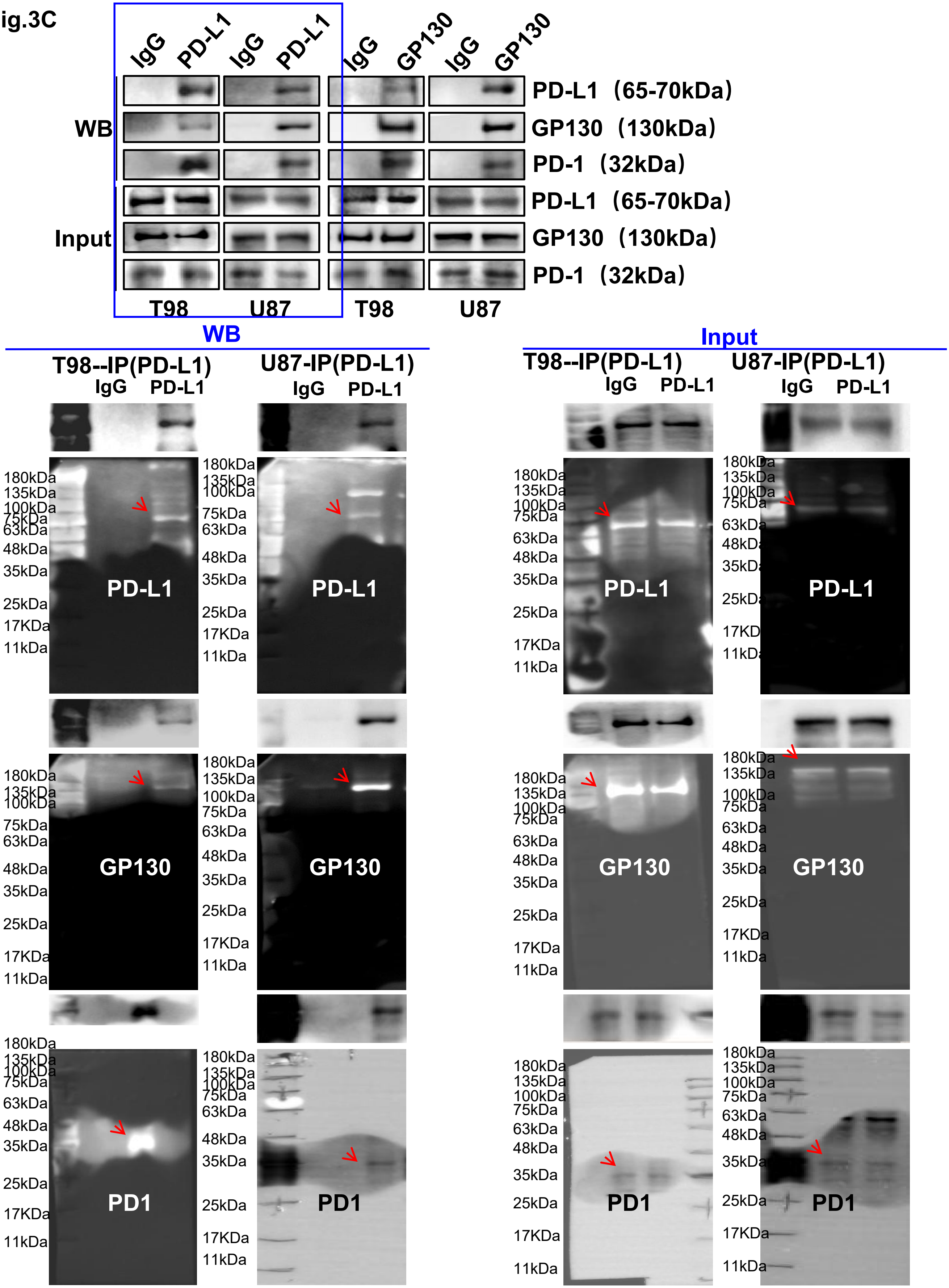
IgG
PD-L1
IgG
PD-L1
IgG
GP130
IgG
GP130
WB
Input
PD-L1（65-70kDa）
GP130（130kDa）
PD-1（32kDa）
PD-L1（65-70kDa）
GP130（130kDa）
PD-1（32kDa）
T98
U87
T98
U87
WB
Input
T98--IP(PD-L1)
U87-IP(PD-L1)
T98--IP(PD-L1)
U87-IP(PD-L1)
PD-L1
GP130
PD1

Fig.3C

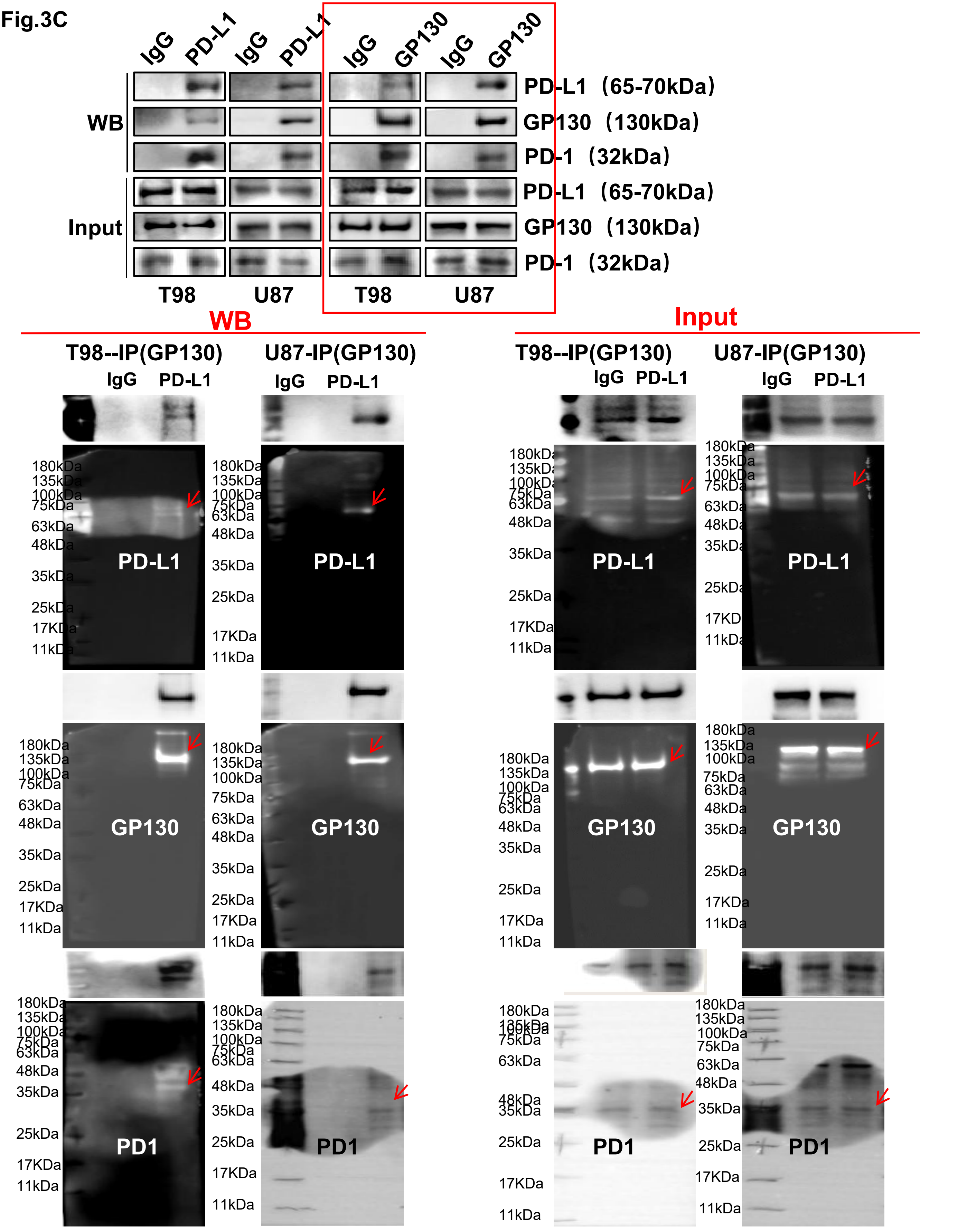
IgG
PD-L1
IgG
PD-L1
IgG
GP130
IgG
GP130
WB
Input
PD-L1 (65-70kDa)
GP130 (130kDa)
PD-1 (32kDa)
PD-L1 (65-70kDa)
GP130 (130kDa)
PD-1 (32kDa)
T98
U87
T98
U87
WB
Input
T98--IP(GP130)
U87-IP(GP130)
T98--IP(GP130)
U87-IP(GP130)
IgG
PD-L1
PD-L1
GP130
PD1
180kDa
135kDa
100kDa
75kDa
63kDa
48kDa
35kDa
25kDa
17KDa
11kDa

Fig.3E

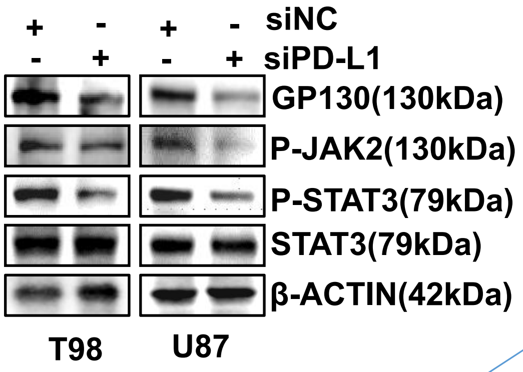

Fig.2A T98 blot was examined for
1） PD-L1
2) β-ACTIN
3） GP130(placed in Fig.3E: T98--GP130)

T98

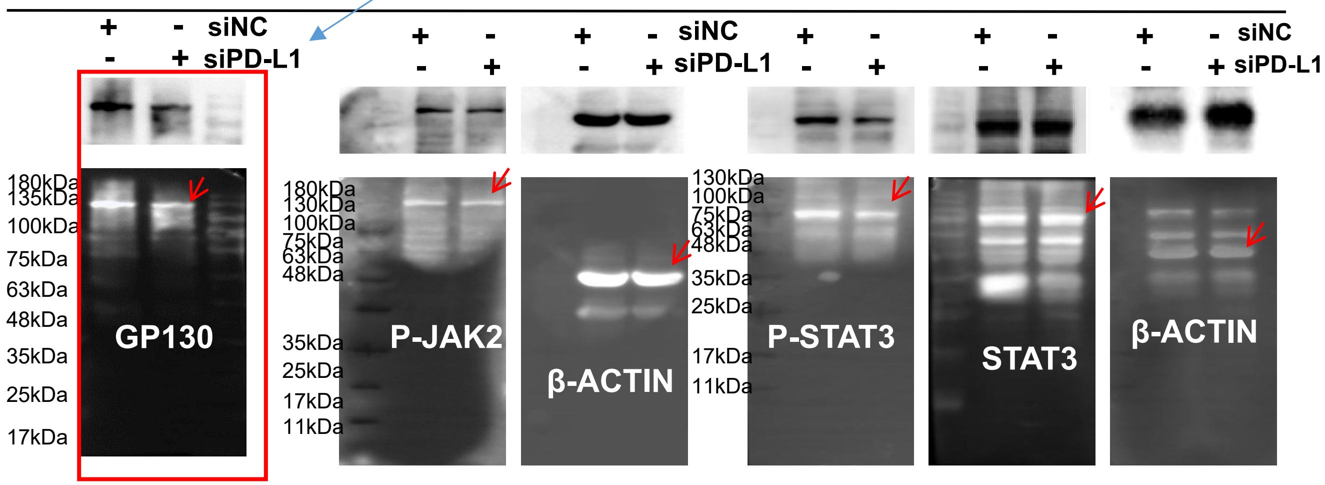

U87

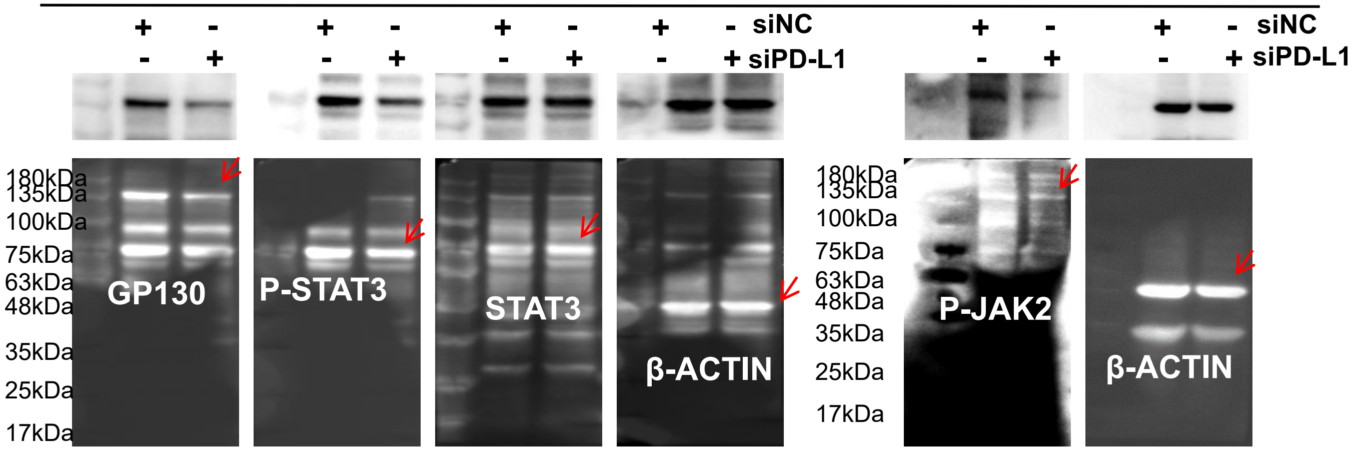

Fig.3F

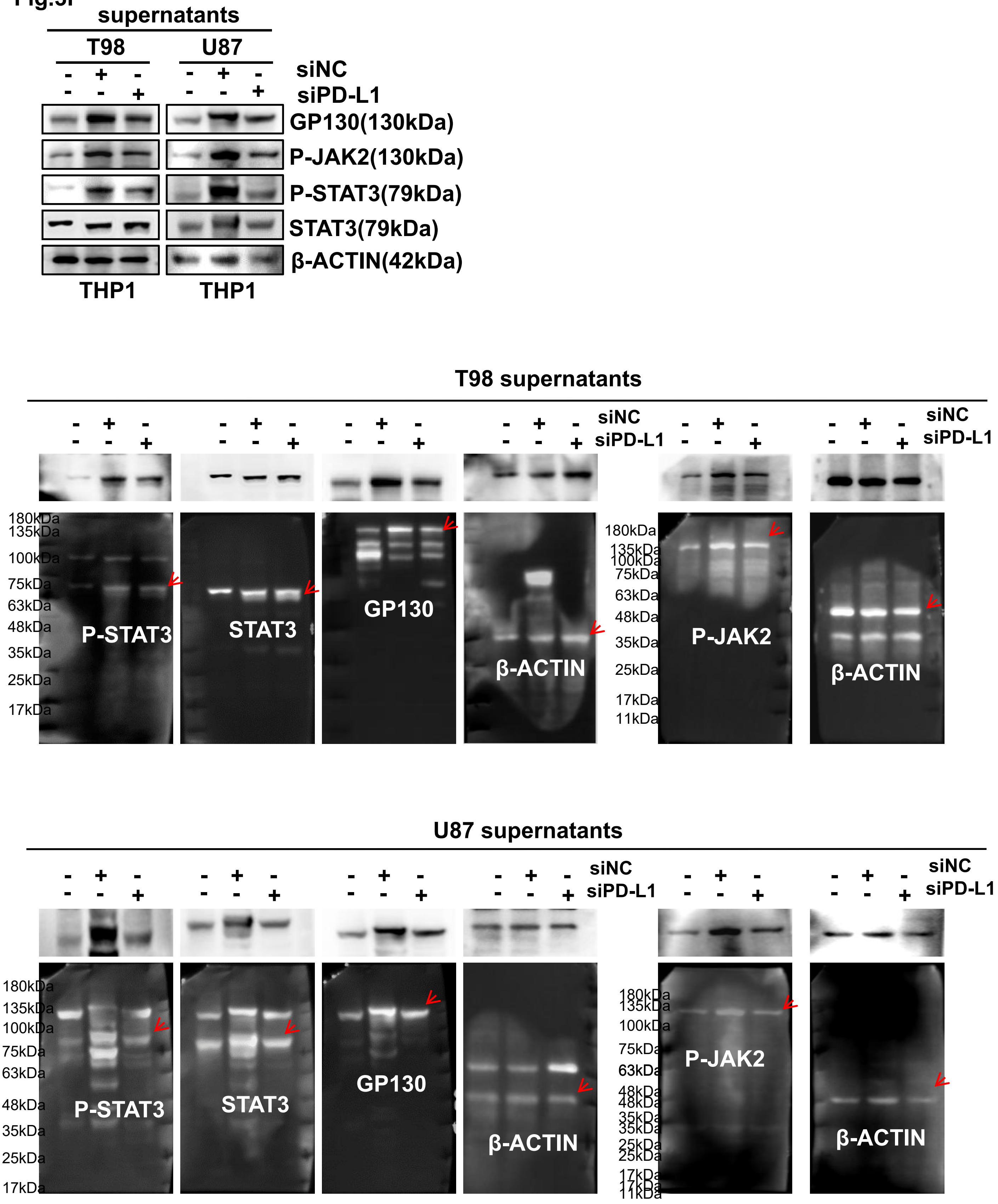

supernatants
T98
U87
siNC
siPD-L1
GP130(130kDa)
P-JAK2(130kDa)
P-STAT3(79kDa)
STAT3(79kDa)
β-ACTIN(42kDa)
THP1
THP1
T98 supernatants
siNC
siPD-L1
180kDa
135kDa
100kDa
75kDa
63kDa
48kDa
35kDa
25kDa
17kDa
11kDa
P-STAT3
STAT3
GP130
β-ACTIN
P-JAK2
β-ACTIN
U87 supernatants
siNC
siPD-L1
P-STAT3
STAT3
GP130
β-ACTIN
P-JAK2
β-ACTIN

Fig.4A

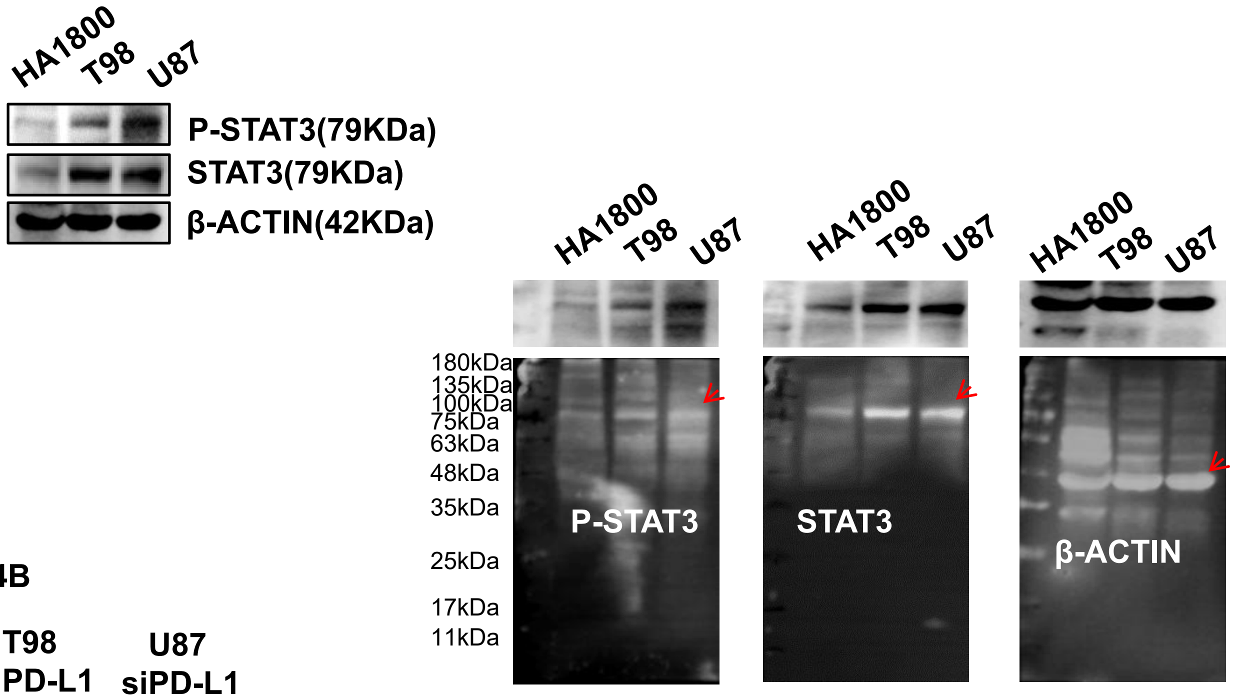
HA1800
T98
U87
P-STAT3(79KDa)
STAT3(79KDa)
β-ACTIN(42KDa)
180kDa
135kDa
100kDa
75kDa
63kDa
48kDa
35kDa
25kDa
17kDa
11kDa
P-STAT3
STAT3
β-ACTIN

Fig.4B

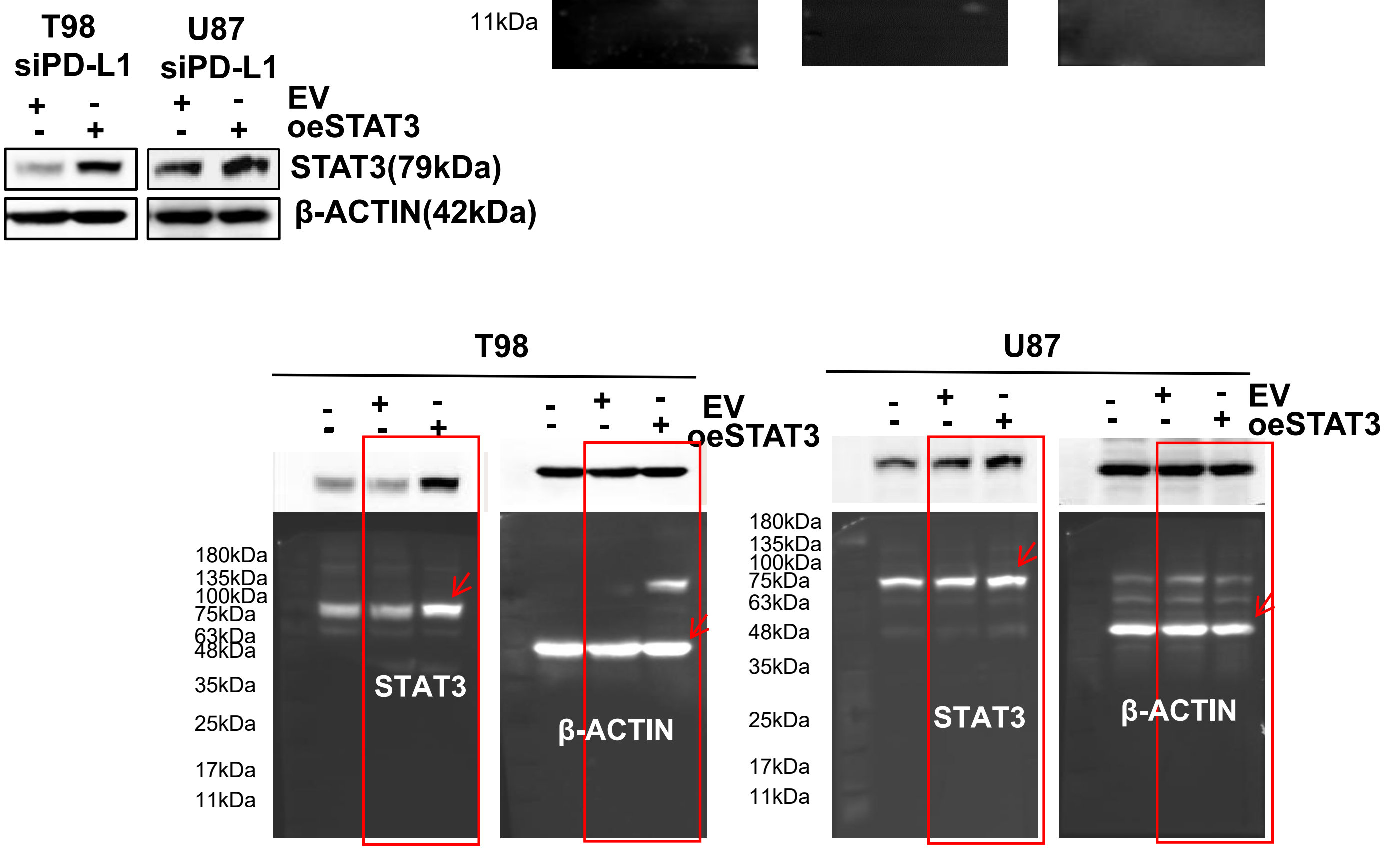
T98
siPD-L1
U87
siPD-L1
EV
oeSTAT3
STAT3(79kDa)
β-ACTIN(42kDa)
T98
U87
180kDa
135kDa
100kDa
75kDa
63kDa
48kDa
35kDa
25kDa
17kDa
11kDa
STAT3
β-ACTIN

Fig.5D

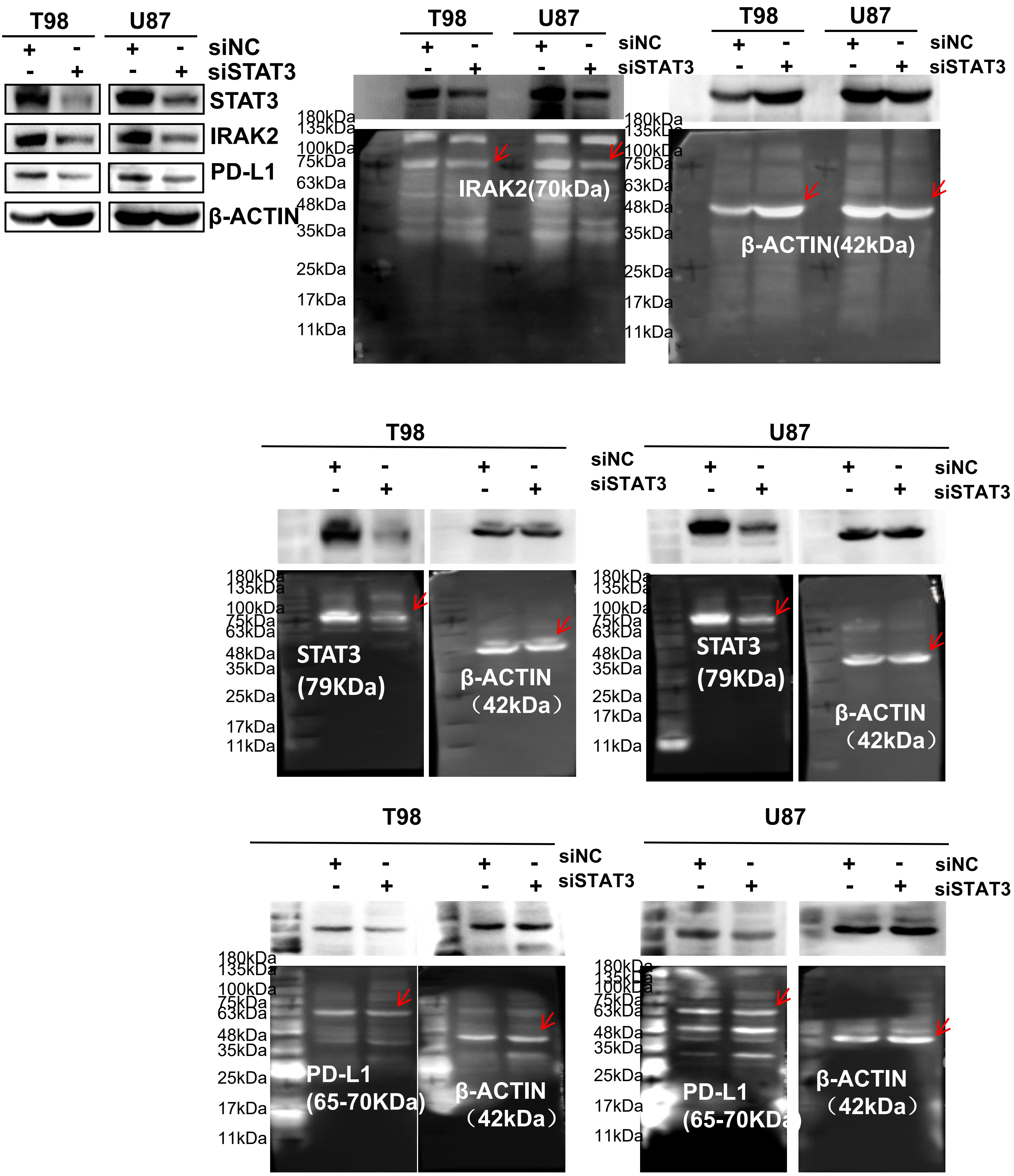
T98
U87
siNC
siSTAT3
STAT3
IRAK2
PD-L1
β-ACTIN
IRAK2(70kDa)
β-ACTIN(42kDa)
STAT3 (79KDa)
β-ACTIN (42kDa)
PD-L1 (65-70KDa)
180kDa
135kDa
100kDa
75kDa
63kDa
48kDa
35kDa
25kDa
17kDa
11kDa

Fig.5F

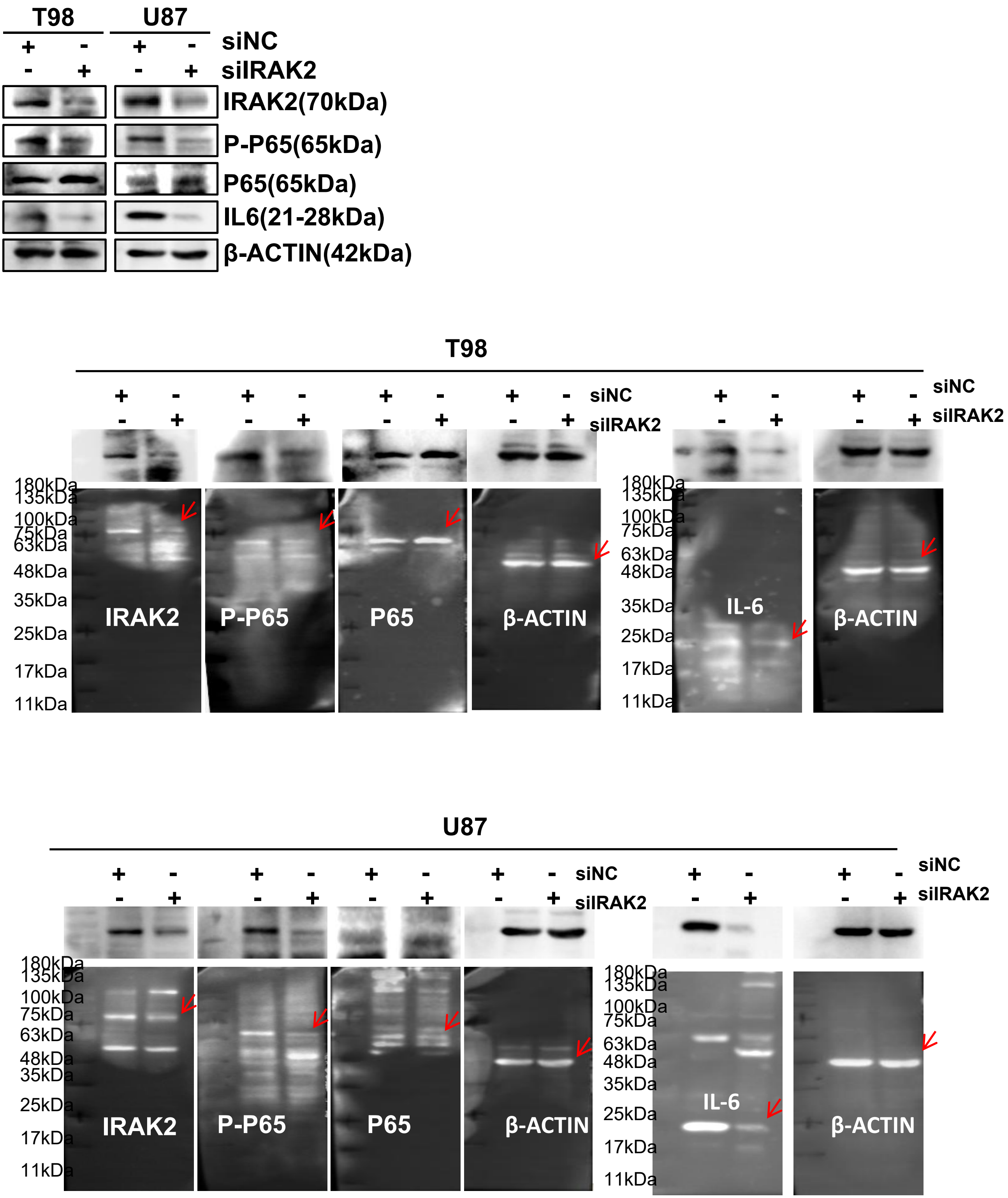
T98
U87
siNC
siIRAK2
IRAK2(70kDa)
P-P65(65kDa)
P65(65kDa)
IL6(21-28kDa)
β-ACTIN(42kDa)
T98
siNC
siIRAK2
180kDa
135kDa
100kDa
75kDa
63kDa
48kDa
35kDa
25kDa
17kDa
11kDa
IRAK2
P-P65
P65
β-ACTIN
IL-6
β-ACTIN
U87
siNC
siIRAK2
IRAK2
P-P65
P65
β-ACTIN
IL-6
β-ACTIN

Fig.6A

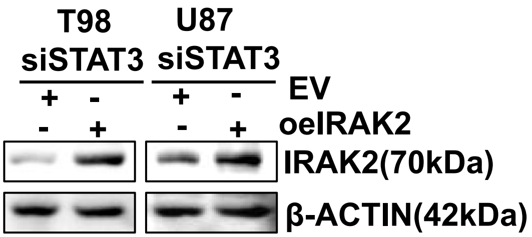
T98
siSTAT3
U87
siSTAT3
+ - + - EV
- + - + oeIRAK2
IRAK2(70kDa)
β-ACTIN(42kDa)

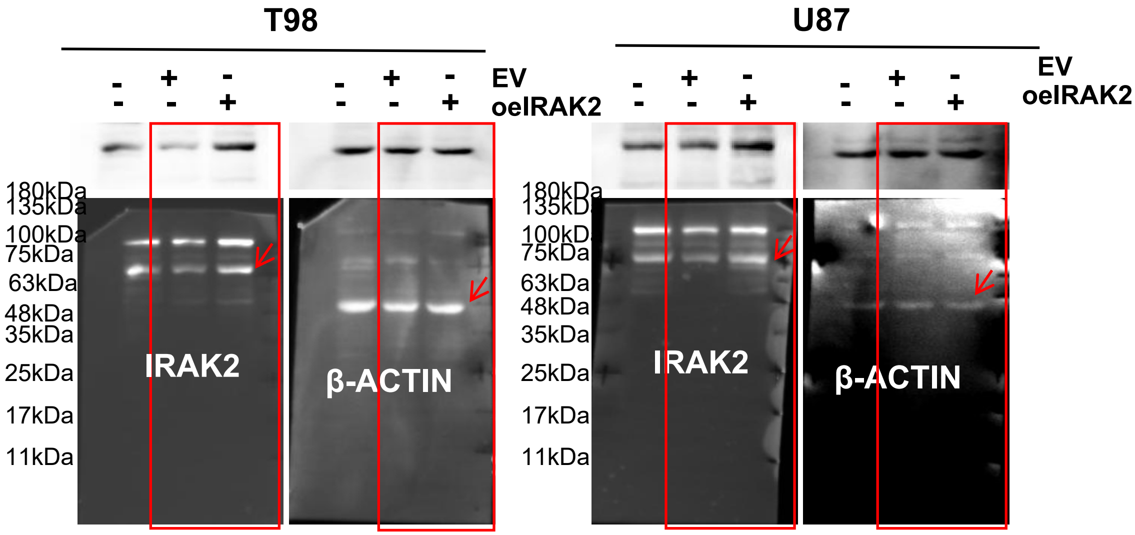
T98
U87
EV
oeIRAK2
180kDa
135kDa
100kDa
75kDa
63kDa
48kDa
35kDa
25kDa
17kDa
11kDa
IRAK2
β-ACTIN

Fig. 7A

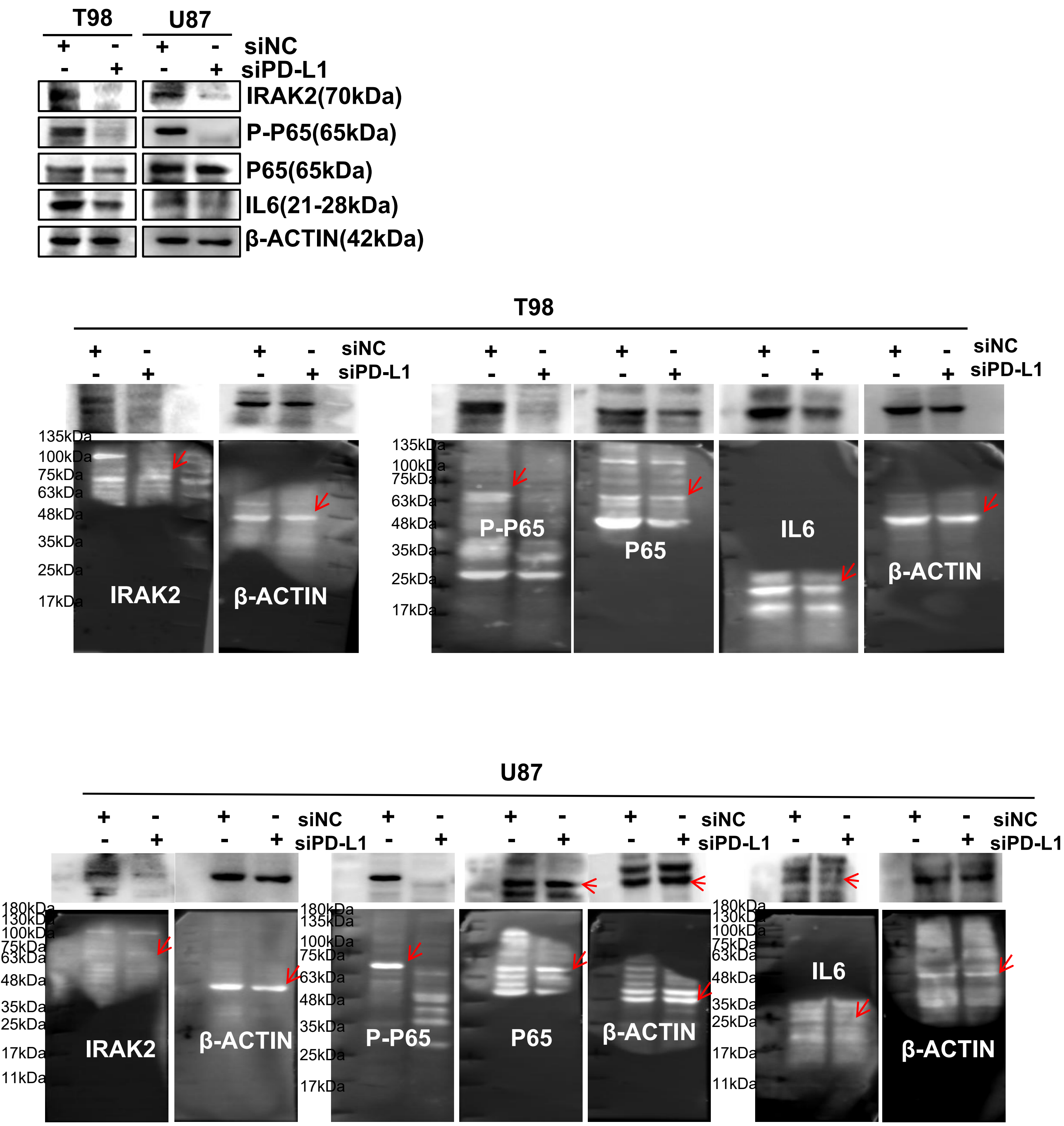
T98
U87
siNC
siPD-L1
IRAK2(70kDa)
P-P65(65kDa)
P65(65kDa)
IL6(21-28kDa)
β-ACTIN(42kDa)
T98
siNC
siPD-L1
135kDa
100kDa
75kDa
63kDa
48kDa
35kDa
25kDa
17kDa
IRAK2
β-ACTIN
P-P65
P65
IL6
β-ACTIN
U87
siNC
siPD-L1
180kDa
130kDa
100kDa
75kDa
63kDa
48kDa
35kDa
25kDa
17kDa
11kDa
IRAK2
β-ACTIN
P-P65
P65
β-ACTIN
IL6
β-ACTIN

Supplementary Figure 4

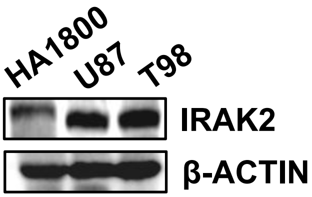

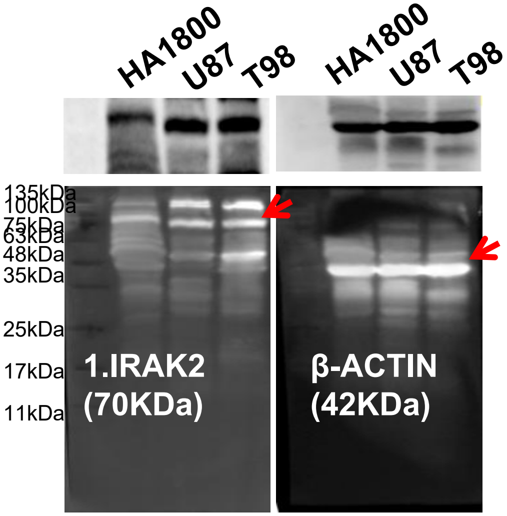

Supplement: Supplementary file 2 — Supplementary Material 2 [file 41598_2025_19169_MOESM2_ESM.pdf]
