# Supplementary material for: PD-L1 autoregulation promotes the proliferation, migration and invasion of glioblastoma cells via GP130/JAK2/STAT3/IRAK2/IL6 signaling pathway
Source: Sci Rep. 2025 Oct 8;15:35186. doi: 10.1038/s41598-025-19169-2 (PMC12508128; doi:10.1038/s41598-025-19169-2)
Supplement: Supplementary file 3 — Supplementary Material 3 [file 41598_2025_19169_MOESM3_ESM.pdf]

Supplementary Figure 1

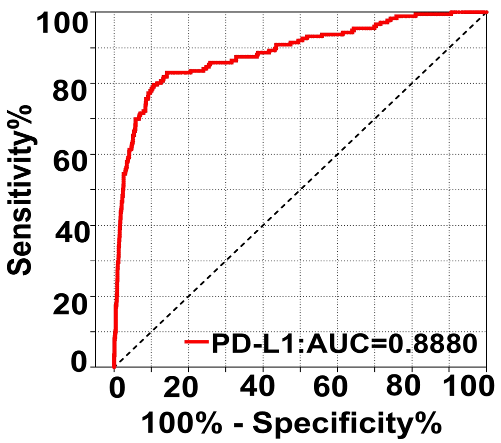

Supplementary Fig.1 ROC curve of PD-L1 ROC curve of PD-L1 RNA from TCGA-GBM dataset. GBM samples (n=176) and normal controls (n=1152).

Supplementary Figure 2

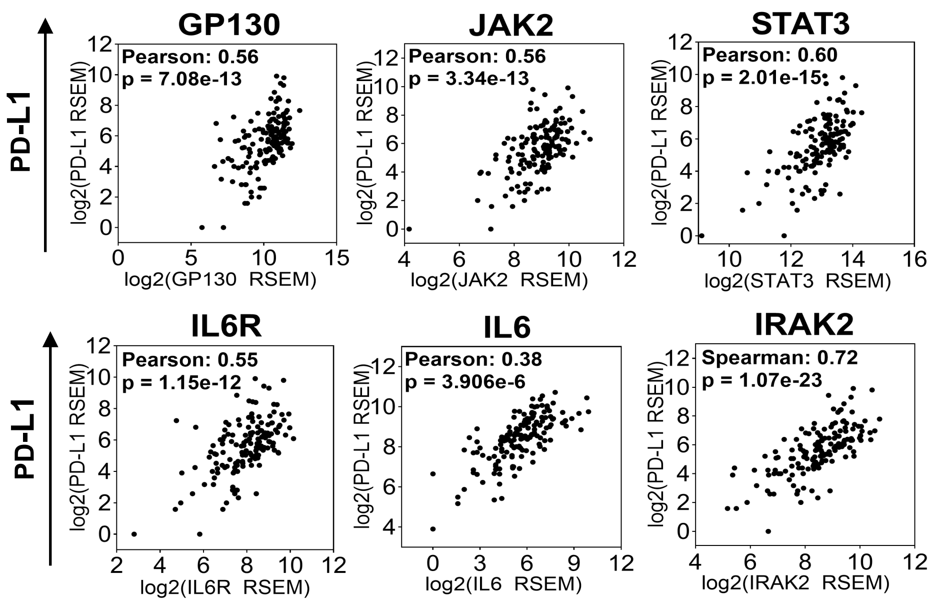

Supplementary Fig.2 The associations of PD-L1 with GP130/JAK2 signaling in GBM The Spearman association data was downloaded from TCGA-GBM dataset in cbioportal.

Supplementary Figure 3

A

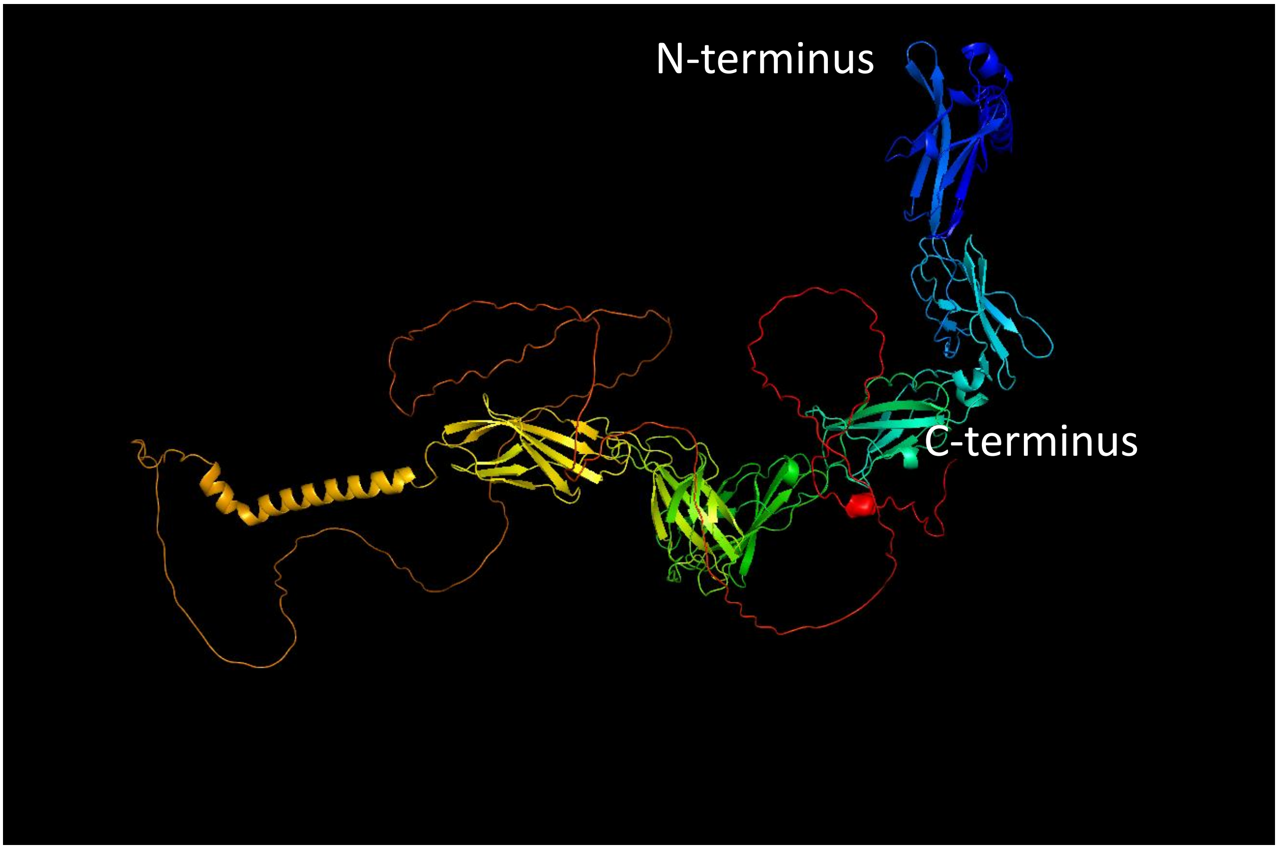

B

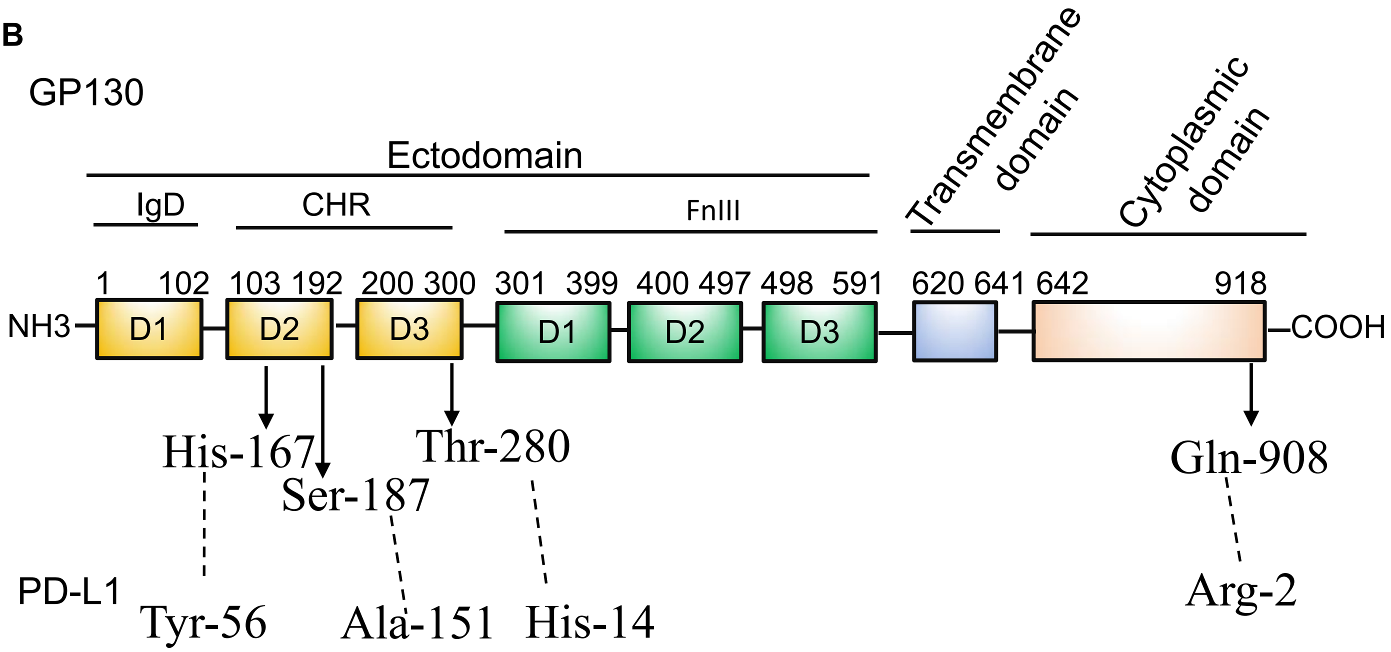

Supplementary Fig.3 The binding between PD-L1 and GP130 (A)PD-L1 interacted with GP130. (B) Amino acid residues in PD-L1 interacted with D2~D3 domains of GP130.

Supplementary Figure 4

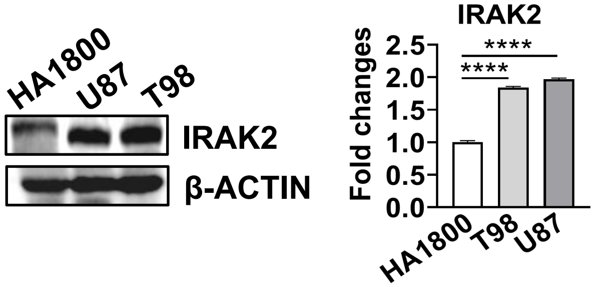

Supplementary Fig.4 IRAK2 expression in HA1800 cell line and GBM cell lines

Supplementary Figure 5

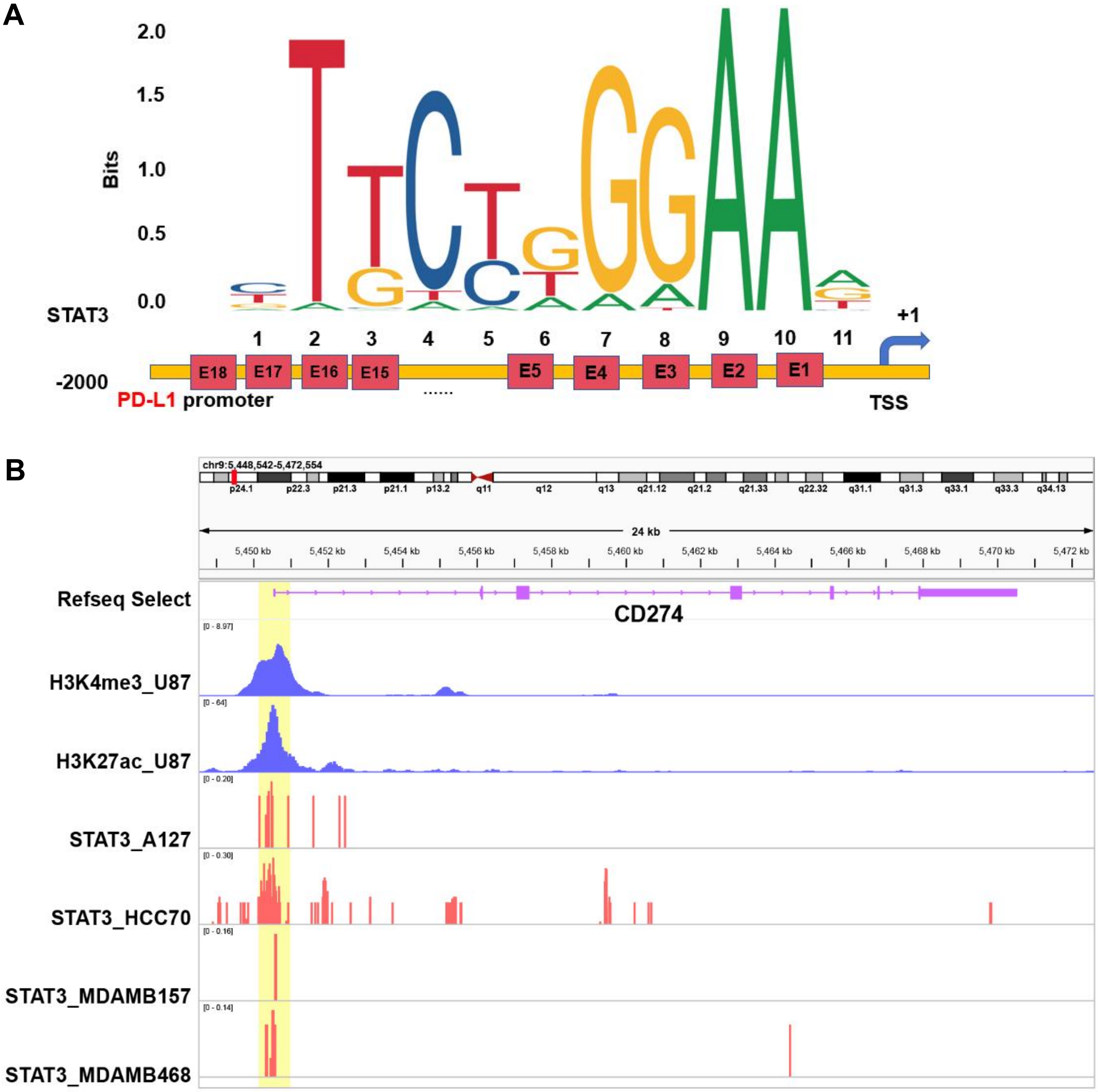

Supplementary Figure 5 Analysis of STAT3 binding to PD-L1 promoter
(A) Potential STAT3 bound sites on PD-L1 promoter. JASPAR was used to predict the possible binding sites. (B) ChIP-seq peak analyses of H3K4me3, K3K27ac and STAT3 on PD-L1 (also named as CD274) promoter.

Supplementary Figure 6

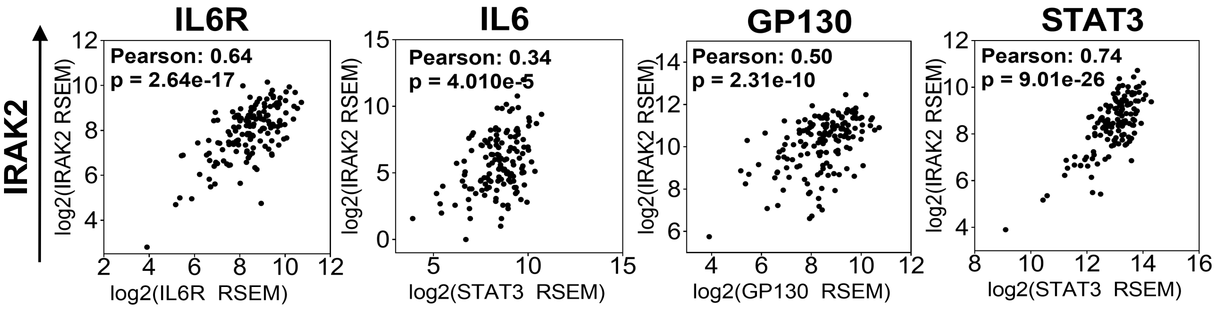

Supplementary Fig.6 The associations of IRAK2 with IL6R, IL6, GP130 and STAT3 in GBM The Spearman association data was downloaded from TCGA-GBM dataset in cbioportal.

Supplementary Figure 7

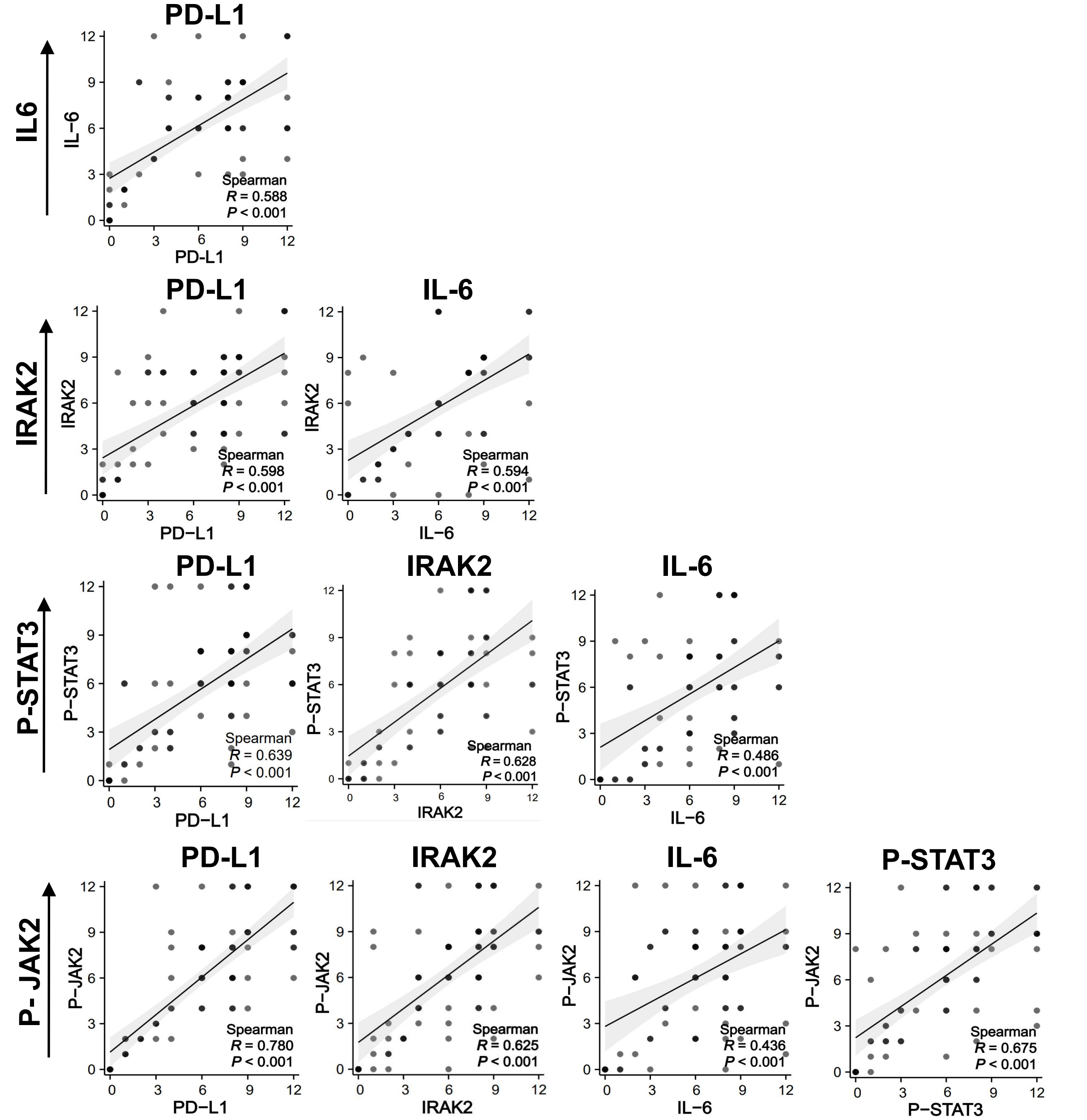

Supplementary Figure 7 Correlations among PD-L1, IL6, IRAK2, P-STAT3 and P-JAK2.

Supplementary Figure 8

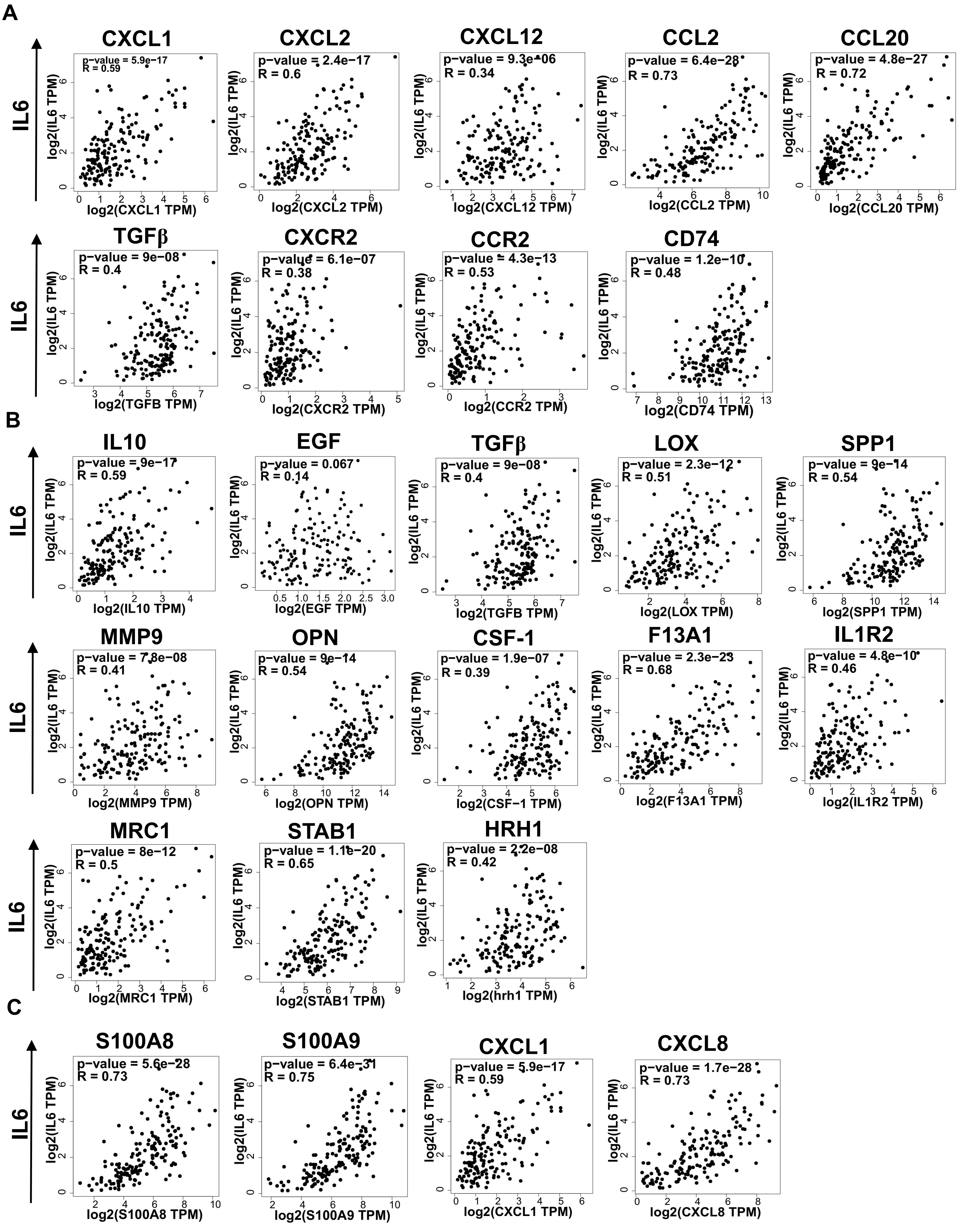

Supplementary Fig.8 The correlations of IL6 with MDSC, macrophage and DC inhibition The Spearman correlation analysis was performed on TCGA-GBM dataset using GEPIAII. (A)The correlations of IL6 with MDSC-associated genes. (B)The correlations of IL6 with macrophage markers. (C)The correlations of IL6 with dendrtic cell inhibition-associated genes.
